# Occupational safety and health risks among plumbers: A qualitative study of workplace hazards, safety practices, and system-level gaps in Texas

**DOI:** 10.1371/journal.pone.0356512

**Published:** 2026-08-17

**Authors:** Mary Conger, Avishek Choudhury

**Affiliations:** Industrial and Management Systems Engineering, Benjamin M. Statler College of Engineering and Mineral Resources, West Virginia University, Morgantown, West Virginia, United States of America; Universiti Kebangsaan Malaysia, MALAYSIA

## Abstract

**Background:**

The plumbing trade exposes workers to ongoing workplace hazards that pose significant health risks. This issue is well known but remains unresolved. A key question remains: why have current safety measures, such as training programs, regulatory standards, and personal protective equipment (PPE) requirements, failed to reduce injury rates or significantly improve safety in the plumbing field.

**Objectives:**

Despite well-documented hazards in plumbing work, qualitative research examining why existing safety measures continue to fail plumbers—particularly in trade-specific U.S. contexts such as Texas—remains limited. This study examined the perspectives and daily experiences of 23 Texas plumbers to understand workplace hazards, safety practices, regulatory oversight, and potential ways to improve occupational health, contributing worker-centered qualitative evidence to a field often dominated by administrative statistics or broader construction-industry analyses.

**Methods:**

This qualitative descriptive study used semi-structured interviews (30–45 minutes) with 23 plumbers from various sectors of the Texas plumbing industry, conducted January 24 to March 1, 2025, and analyzed using Braun and Clarke's reflexive thematic analysis.

**Results:**

Thematic analysis identified seven themes, most prominently barriers to PPE use, an entrenched “get-it-done” safety culture, and gaps in policy enforcement and quality control, as well as challenges in tool and equipment safety, training adoption, and physical safety and insurance protections. These themes revealed differing views between plumbers and organizational stakeholders, examined through structural, cultural, and behavioral lenses.

**Conclusion:**

Within this qualitative sample, system weaknesses, unsafe practices, and adverse health outcomes appeared closely intertwined among Texas plumbers. Closing the perceptual gap between plumbers and employers may require coordinated efforts from both groups, offering a foundation—rather than a generalizable model—for future training, research, and policy development. Objectives: Despite well-documented hazards in plumbing work, qualitative research examining why existing safety measures continue to fail plumbers—particularly in trade-specific U.S. contexts such as Texas—remains limited. This study examined the perspectives and daily experiences of 23 Texas plumbers to understand workplace hazards, safety practices, regulatory oversight, and potential ways to improve occupational health, contributing worker-centered qualitative evidence to a field often dominated by administrative statistics or broader construction-industry analyses. Methods: This qualitative descriptive study used semi-structured interviews (30–45 minutes) with 23 plumbers from various sectors of the Texas plumbing industry, conducted January 24 to March 1, 2025, and analyzed using Braun and Clarke's reflexive thematic analysis. Results: Thematic analysis identified seven themes, most prominently barriers to PPE use, an entrenched “get-it-done” safety culture, and gaps in policy enforcement and quality control, as well as challenges in tool and equipment safety, training adoption, and physical safety and insurance protections. These themes revealed differing views between plumbers and organizational stakeholders, examined through structural, cultural, and behavioral lenses. Conclusion: Within this qualitative sample, system weaknesses, unsafe practices, and adverse health outcomes appeared closely intertwined among Texas plumbers. Closing the perceptual gap between plumbers and employers may require coordinated efforts from both groups, offering a foundation—rather than a generalizable model—for future training, research, and policy development.

## 1. Introduction

Plumbing is fundamental to public health and building infrastructure. It is often seen as a high-risk occupation [1]. Workers face physical dangers (falls and injuries), encounter chemical hazards (sewer gas), and ergonomic issues from repetitive tasks. Despite these deaths or injuries, plumber-specific qualitative research remains limited compared with the broader construction safety literature. Studies more often focus on the construction sector in general [2] rather than on plumbing safety specifically. In standard occupational descriptions, plumbers and related trades lift heavy materials, climb ladders, and work in tight spaces; outdoor work “in all types of weather” may be required; and common injuries include cuts, burns, and ladder falls [3]. These day-to-day conditions map directly onto a broad hazard taxonomy relevant to occupational safety and health: physical hazards (slips, trips, falls; struck-by; hot-work burns), ergonomic hazards (heavy lifting, sustained kneeling/crouching in cramped areas, overhead force), chemical hazards (sewer gases such as hydrogen sulfide; cleaning chemicals), biological hazards (sewage contact and aerosolized water risks), respiratory hazards (fumes, gases, and oxygen-deficient atmospheres), electrical hazards (energized work and wet environments), and psychosocial hazards (on-call emergency work, time pressure, subcontracting and small-business dynamics) [3].

The available prevalence measures for plumbers in the U.S. primarily come from administrative injury surveillance rather than direct exposure monitoring. Although these data do not specify the precise tasks (e.g., sewer line replacement vs residential fixture installation), the injury “signature” strongly suggests that ergonomic load handling and awkward postures are central mechanisms of harm in this trade group [4]. International primary research reinforces that musculoskeletal disorders can dominate plumbers’ health burden in settings with high manual material handling: in a cross-sectional study of Nigerian plumbers (n = 130), 12‑month prevalence of work-related musculoskeletal disorders was 84.6% and 7‑day prevalence was 50.8%, with low-back, neck, and knee symptoms most common [5]. While transferability to U.S. contexts is uncertain, these findings underscore that musculoskeletal risk plausibly remains high whenever time pressure and physical constraints limit mechanical aids and safe postures [5].

Confined spaces and sewer-related atmospheres make plumbing distinct from many other construction trades. Hazardous atmospheres, engulfment potential, constricting configurations, unguarded machinery, exposed live wires, or heat stress can all be present in typical plumbing spaces such as manholes, vaults, pits, and pipelines [6]. Among chemical hazards, the Occupational Safety and Health Administration (OSHA) identifies hydrogen sulfide as one of the leading causes of workplace gas inhalation deaths and reports 46 worker deaths attributed to hydrogen sulfide during 2011–2017 [7]. A U.S. Department of Labor investigation described a June 2023 incident in which a worker entered a manhole to address a sewer-line blockage, fell about 20 feet, and died following both fall trauma and exposure to a high hydrogen sulfide concentration (1,910 ppm measured by responders) [8]. This workforce is also exposed to biological and respiratory risks and is undercounted when illness does not result in workers’ compensation or employer-reported cases. However, primary infectious disease surveillance suggests that building-water-related pathogens may be especially relevant. A U.S. study using Supplemental Legionnaires’ Disease Surveillance System data from 39 jurisdictions (2014–2016) found an elevated incidence in the construction occupation group compared with other occupations (incidence rate ratio, 1.82; construction n = 269 among cases with known occupation group) [9]. Heat and electrical hazards further compound risk, especially in Texas. OSHA notes that millions of workers are exposed to heat at work; thousands become sick annually, and some cases are fatal, with a large fraction of outdoor fatalities occurring during the first days of exposure when workers are not acclimatized [10]. Texas-specific public health surveillance from Texas Department of State Health Services includes a 2023 regional heat-related illness surveillance report based on emergency department syndromic surveillance: for Central Texas, August–September temperatures were maintained in an 86–110°F range, with 2023 described as the second-hottest summer on record; the report explicitly cautions that the query used (including “dehydration”) may overestimate or underestimate heat-related illness burden [11]. While these are not plumber-only data, they establish an empirically documented environmental context in which outdoor and attic/crawlspace plumbing work may plausibly elevate heat risk beyond what is typical in milder climates. Electrical risks are also salient in plumbing because water, powered tools, and energized building systems can co-occur. OSHA emphasizes that electrical injuries frequently arise from contact with power lines, lack of ground-fault protection, missing or discontinuous grounding paths, equipment misuse, and improper extension-cord practices [12].

This study aims to qualitatively explore the safety risks associated with plumbing and identify the barriers that prevent effective risk management. In this study, we focus on the state of Texas in the United States. A Texas focus is justified on multiple empirical and structural grounds. Texas ranks among the states with the highest employment of plumbers, pipefitters, and steamfitters (41,890 employed in May 2023) [13]. Texas is 40.3% Hispanic/Latino, 17.6% foreign-born, and 35.1% of residents aged 5+ speak a language other than English at home, demographics with direct implications for training access, safety communication, and perceived vulnerability in reporting systems [14]. The regulatory environment adds another layer: the Office of the Texas Governor [4] states that Texas workers’ compensation is not mandatory for employers (except in much of the public sector), meaning the incentive and support structures for injury reporting and treatment can differ from those in mandatory coverage states [15].

## 2. Methods

### 2.1 Ethics statement

The West Virginia University Institutional Review Board (IRB) approved Protocol #2407010354, ensuring the integrity of this study. An exemption was granted under 45 CFR 46.101(b)(2) because the interviews were anonymous. This exemption was justified since the research posed no risk to the participants’ financial standing, employability, or reputation [16]. To ensure informed participation, each of the 23 plumbers received a detailed cover letter outlining the study’s goals, procedures, the voluntary nature of their involvement, and the safeguards in place to protect their confidentiality. All participants provided verbal consent to confirm their understanding and agreement. No personally identifiable information was collected, thereby protecting their privacy throughout the study and ensuring adherence to ethical standards.

### 2.2 Study context

This inquiry took place in Texas, which has about 45,000 licensed plumbers across various skill levels (Tradesman, Journeyman, and Master) and an estimated total of 74,000, including apprentices and inspectors [17,18]. Data collection took place in various work environments, including active construction sites, commercial buildings, and office locations. Topics varied, including confined spaces such as crawlspaces, attics, and trenches. The Occupational Safety and Health Administration [19] identifies these areas as high-risk zones where hazards such as falls, tool injuries, and exposure to hazardous substances are common. This context is crucial for understanding the safety challenges plumbers face in Texas.

### 2.3 Methodological framework

This qualitative descriptive study investigated Texas plumbers’ experiences of safety and health risks using an inductive, participant-centered design that allowed personal narratives to direct the research. To reduce potential bias, the researcher engaged in reflexive bracketing, consciously setting aside preconceived notions about issues such as tool misuse or PPE use. A reflexive journal was kept throughout the study to document and interrogate these assumptions, supporting a focus on participants’ own perspectives [20]. The analysis followed Braun and Clarke's [21,22] reflexive thematic analysis framework, which provided a structured yet flexible method for identifying and interpreting patterns across the interview data.

### 2.4 Participants

Participants comprised actively licensed/registered plumbers working in Texas, including owners and employees. It represented all licensure levels recognized by the Texas State Board of Plumbing Examiners (TSBPE). Participants were employed across diverse sectors, including residential service and repair, commercial construction and retrofit, industrial and institutional maintenance, and all applicable plumbing industries/operations. Work settings regularly involved high-risk environments such as confined spaces (crawlspaces, attics, lift stations, and manholes), elevated locations, trenches, and areas with exposure to sewage, silica dust, and hazardous gases. Preference was given to those with firsthand experience of injuries or near-misses, aligning with the study’s focus on plumbing safety. The following criteria allow participants to be interviewed:

- 18 years of age and older
- Active participation in the plumbing trade
- Licensed, endorsed, or registered with an apprenticeship card or higher with the TSBPE
- Active participation in Texas in the plumbing trade

Interviews continued until data saturation was reached: new codes generated per interview were tracked throughout data collection, and interviewing stopped once several consecutive transcripts introduced no additional codes, consistent with the emergence of repeated themes and the absence of new insights in subsequent sessions [21].

### 2.5 Data collection and survey guide

Recruitment drew on a dataset of licensed Texas plumbers who had completed continuing education courses; a recruitment statement was distributed to this group inviting participation, and interviews were scheduled with plumbers who volunteered and regularly performed tasks involving workplace hazards. All participants provided voluntary, informed consent before participating in the study to ensure their ethical involvement. In-depth data were collected through semi-structured interviews conducted via Zoom between January 24 and March 1, 2025. Each interview lasted about 30–45 minutes. Interviews aimed at capturing plumbers’ experiences regarding occupational safety. Table 1 shows the interview guide. The guide was pilot-tested with two non-participating plumbers to ensure clarity and relevance. Minor adjustments were made to enhance the clarity of question phrasing.

**Table 1:** Interview guide.

Leading Questions	Probing Questions
**Before beginning, the interviewer confirmed: “This interview is confidential. Your name will not be used in any publication, and you may decline to answer any question or stop at any time.”**	**None**
For Employees	
1. For the record, please tell us a little about yourself and what your license level is. Can you share your age?	None
2. Can you tell me about a time in which you experienced an on-the-job injury (i.e., contact with an object [including struck-by], overexertion/bodily reaction, and/or fall, slip, or trip)?	a. Walk me through that day that you experienced this injury regarding the above topics? b. What was the nature of the injury, and did it require medical treatment? Please explain in detail. Were you tired? From overworking or the workload? Do you believe fatigue played a role? c. Was this injury reported to your supervisor or anyone at your company? Please explain in detail. d. Did you receive training on this subject previously? If so, please explain the type of training, including its length and format, as well as any course details you remember or would like to change/alter. e. After the injury, can you describe the atmosphere at work (i.e., were there changes to working conditions or a shift to a more safety-minded environment)? f. Have you experienced near misses? g. Can you share about PPE? Do you use it? What about tools, are they in good condition/quality or older?
For Owners	
3. Do you feel your employees report accidents readily?	Why or why not? Are they worried about reporting? Are they comfortable with reporting?
4. Do you require your staff to wear PPE and use it?	None

### 2.6 Interviewer

The researcher’s 30 years of experience as a licensed Texas plumber and 11 years as an OSHA-authorized instructor shaped the interview process. This background enabled targeted inquiries into plumbing-specific hazards, such as tool misuse and confined-space risks. Reflexive practices ensured that this expertise did not overshadow participants’ voices. A reflexive journal was maintained to record the researcher’s thoughts and potential biases, thereby supporting the commitment to epoché by bracketing preconceptions [20]. For example, one journal entry noted: “During Participant 1's account of the boiler falling on him, I noticed I wanted to press harder because I've been taught that exact scenario in OSHA classes about using mechanical means to move heavy items. I held back and let him finish his own reasoning before asking a neutral follow-up.” Another entry recorded a similar moment of restraint: “During Participant 10's account of skipping assessment of the condition of the ladder he borrowed, I noticed I wanted to press harder because of the statistics of ladder injuries from that exact scenario in OSHA classes. I held back and let him finish his recount.” A third entry reflected a different kind of restraint, driven by sensitivity to the participant's experience rather than professional expertise: “During Participant 19's account of his eye injury, I noticed I wanted to press harder, but this was a very sensitive occurrence, and I held back due to the nature of the injury and loss of his sight/use in that eye.” Together, these excerpts illustrate bracketing as an active practice during data collection, prompted by both professional expertise and emotional sensitivity to participants’ experiences, rather than only a procedural claim. [20]

### 2.7 Data analysis

During the interviews, the researcher employed active listening techniques. This included paraphrasing and asking clarifying questions to capture participants’ perspectives without bias. This approach created a conversational environment where participants felt at ease sharing sensitive experiences. Unreported injuries or workplace pressures exemplify this. All interviews were audio- and video-recorded with participants’ explicit consent. This adhered to the ethical guidelines set by the U.S. Department of Health and Human Services [16]. Participants were informed that the recordings would be used solely for transcription and analysis. Further, participants were told that all data would be anonymized. To ensure confidentiality, recordings were stored on a secure, IRB-compliant cloud server that was accessible only to the research team. Transcripts were prepared promptly after each interview. Zoom’s transcription tool served as a starting point, followed by manual verification to correct errors and remove identifying details, such as names or specific job sites. The transcription process sought to capture participants’ words verbatim. This includes pauses and emotional cues to retain the essence of their narratives.

Data analysis followed Braun and Clarke's [21] six-phase thematic analysis framework to identify and interpret patterns in the participants’ accounts. The researcher was immersed in the data by transcribing interviews and rereading the transcripts. It was noted that their initial thoughts were to gain a solid understanding of the situation. Recordings were securely stored on a cloud server that met IRB requirements. The initial transcripts were checked for accuracy using Zoom’s transcription tool. Personal identifiers, such as names and locations, are used to protect anonymity. Second, an inductive coding process was used to label text segments related to injuries, tools, PPE, environmental factors, and organizational issues. This process used Microsoft Word and qualitative software, yielding codes such as “tool misuse” and “lack of PPE.” This process helped mitigate bias and ensured the codes reflected the participants’ stories. Third, the codes were grouped into potential themes based on recurring patterns. For example, the terms “no gloves” and “no safety glasses” were combined into a non-compliance theme for PPE. The team met via Zoom to discuss and refine these themes, ensuring they captured the full scope of the data. Fourth, themes were cross-checked against coded excerpts and the entire dataset for consistency and relevance. Adjustments were made as needed, such as merging the ‘fall’ and ‘trip’ codes into a single hazard theme. Fifth, the researcher defined and named the themes to reflect their importance. The researcher selected representative quotes to connect the findings with participants’ voices, such as “Barriers to Proper Use of PPE” for non-compliance. Finally, in the sixth phase, the researcher wove these themes into a cohesive narrative for the results section. This narrative linked the themes to participants’ accounts and relevant literature while emphasizing the focus on lived experiences. Data saturation was reached when no new themes emerged in the later interviews. Coding was conducted by a single researcher; the research team's Zoom discussions functioned as peer debriefing during theme refinement rather than independent double-coding, and no formal intercoder reliability statistic was calculated [21].

## 3. Results

### 3.1 Participants

Participants were purposefully selected to represent a range of individuals actively engaged in plumbing tasks and exposed to workplace hazards (S1 Table). The final group included 23 plumbers (22 males and one female). Years of experience and employment classification (owner/employee, business size) were elicited through organic follow-up during the interview rather than the fixed interview guide questions. Table 2 represents participant characteristics.

**Table 2:** Participant characteristics (n = 23).

	N (%)
Sex	
Female	1 (4.3)
Male	22 (95.7)
Age Category	
18-35	7 (30.4)
36-55	12 (52.2)
56-65	2 (8.7)
66 and over	2 (8.7)
Years of Experience	
1-5	2 (8.7)
6-10	6 (26.1)
11-20	4 (17.4)
21 and over	11 (47.8)
Business Structure	
Self-Employed (works for themselves)	6 (26.1)
Small Company (less than 10 employees)	8 (34.8)
Large Company (more than 10 employees)	9 (39.1)
Role	
Owner	6 (26.1)
Employee	17 (73.9)

Note: Business Structure and Role are independent classifications (each summing to 100%); a participant coded as an Owner may also be coded as Self-Employed, Small Company, or Large Company.

### 3.2 Themes

The thematic analysis identified seven distinct themes highlighting the safety challenges plumbers face. These themes are detailed in Table 3 and supported by quotes reflecting the participants’ views. The themes align with safety issues noted in other high-risk trades [19,23]. These themes, as illustrated in Table 3, show how the underlying issues are interconnected rather than isolated.

**Table 3:** Themes and illustrative participant quotes.

Theme	Illustrative Quotes
Barriers to Proper Use of PPE	“I wasn’t wearing gloves, and the drill jammed, spun, and broke my wrist” **(Participant 9)**. “No PPE, just soaked in sewage from a manhole” **(Participant 20)**. “I didn’t realize that there was a 3-inch pipe inside, and when I had it like this, it came, and it grabbed my fingers, and it was pretty gnarly, and it was pretty painful” **(Participant 3)**. “I was in regular work clothes, maybe safety glasses, no face mask or gloves” (**Participant 6**). “Some resist, saying they don’t wear gloves on the job” (**Participant 15)**. “The strap snapped back and struck my left eye, detaching the retina” **(Participant 19).**
Challenges in Safe Tool Use	“Dropped a circular saw with the blade locked open, cut my stomach” **(Participant 2)**. “The grinder slipped without a guard, cut my hand” **(Participant 9)**. “We replaced a faulty hammer drill last week… not safe” **(Participant 10)**. “Got his glove caught… cutting off the tip of his finger” **(Participant 15).** “Battery-powered band saws with two-hand controls… reduce risks like cuts” **(Participant 15)**. “Two plumbers injured their fingers using a pro press tool… caused severe swelling.” **(Participant 17).**
Health Impacts of Unsafe Exposure	“Chronic sewer gas exposure caused respiratory problems” **(Participant 5)**. “Got a staph infection from sewer manhole exposure” **(Participant 20)**. “There were some, maybe some questions about some intestinal blockages” **(Participant 1)**. “He ended up passing out due to the heat… had to take him to the emergency room” **(Participant 3)**. “The gas and the smoke inhalation… got me pretty good… my lungs were kind of burning a little bit” **(Participant 4)**. “No air movers… would’ve reduced the risk by improving ventilation” **(Participant 21)**.
Challenges in Training Adoption	“Subcontractor crew got a 3-hour orientation instead of full training” **(Participant 2)**. “No training, just rushing, caused the grinder to slip” **(Participant 9)**. “OSHA classes need better accessibility for non-English speakers” **(Participant 11)**. “Neither of us had formal training on scissor lifts” **(Participant 13)**. “Many apprentices don’t learn safety until… licensing classes” **(Participant 14)**. “Training helps… critical for those without field experience” **(Participant 18)**.
Lack of Policies and Monitoring	“Employer dismissed my staph infection, offered no support” **(Participant 20)**. “No reporting in self-owned business” **(Participant 3)**. “They did not familiarize that crew with any of the other protocols, any of the safety stuff **(Participant 2)**. “I’ve fallen off a ladder twice… because I was working alone” **(Participant 8).** “Glove use is mandatory… termination for non-compliance” **(Participant 12)**. “Didn’t do too much hard checking on how certified people are” **(Participant 13)**. “A large dog bit my butt cheek through my pants, causing bruising and minor bleeding” **(Participant 17).**
Absence of Quality Control	“Faulty duct-taped ladder” **(Participant 10)**. “Modified wire brush drill” **(Participant 15**). “One time, during an above-ceiling inspection on a sprinkler system, I was looking up, tripped, and fell into a hole” **(Participant 7)**. “A trailer came loose because it wasn’t properly secured” **(Participant 16)**. “I underestimated the sheetrock’s weight… my ignorance contributed” **(Participant 22).**
Lack of Physical Safety and Insurance	“Fell through a plywood catwalk onto a bookshelf” **(Participant 18)**. “No compensation for injury” **(Participant 20)**. “The warehouse had no power… water and wires everywhere” **(Participant 14)**. “Crawlspace with standing water and exposed aluminum wiring… electrocution risks” **(Participant 18).** “The company didn’t assist with medical bills or pay me for my time off” **(Participant 21)**. “They covered medical bills but offered no pay for a week off due to the staph infection” **(Participant 23).**

#### 1. Barriers to proper use of PPE.

Many workers were hesitant to wear personal protective equipment. Employers, particularly in small firms, frequently prioritize productivity over safety, further widening this disconnect. These gaps lead to higher accident rates reported by plumbers. Examples are gloves, safety glasses, and masks. This reluctance often arose from practical issues. These include time constraints, concerns about how gear affects task performance, and a lack of awareness of potential hazards. For example, Participant 15 noted resistance to wearing PPE.

Participant 9 suffered an injury when a drill jammed while he was not wearing gloves, demonstrating the immediate consequences of this choice. On the other hand, Participant 20 contracted a staph infection after working unprotected in a sewer manhole, showing long-term health risks. Participant 3 did not have gloves on when dealing with a 3-inch pipe and sustained a hand injury. Participant 6 did not have adequate PPE, such as gloves or a face mask, at a lift station, which is a permitted confined space. Unfortunately, Participant 19 experienced a detached retina and loss of eyesight because of an eye injury from a loose rubber strap. Notably, PPE avoidance was not confined to less experienced workers: Participant 20, a business owner with over three decades in the trade, and Participant 3, in an early-career incident, both went unprotected in comparable circumstances. This pattern suggests PPE noncompliance functions as a shared cultural norm across experience levels rather than a knowledge gap concentrated among newer workers. These cases reveal a workplace culture that prioritizes speed and productivity over safety, highlighting the need for education and support to address these challenges.

#### 2. Challenges in safe tool use.

Injuries related to tool use were common and often resulted from improper handling, disabling safety features to work faster, or rushing due to tight deadlines. Participant 2 suffered a stomach laceration when he dropped a circular saw with its safety blade locked open, a clear example of misuse. In contrast, Participant 9 cut his hand on a grinder that was missing its guard, highlighting maintenance issues. Plumbers often view safety measures as impractical, as illustrated by Participant 9’s injury while rushing with a grinder, an incident underscoring the tension between safety compliance and productivity demands. Another severe injury from improper use of tools occurred, resulting in a tip of a finger being cut off (Participant 15). Participant 17 described an injury from a hydraulic crimping tool crushing or swelling a finger. Further, Participant 10 explained finding a faulty hammer drill and the importance of checking hand tools. Participant 15 explained that improvements in tool safety stem from advances in tool design (e.g., two-hand controls, enclosed blades) that reduce risks. These incidents cluster into two distinct mechanisms: behavioral shortcuts under time pressure (Participants 2 and 9, both linked to rushing) and equipment that had degraded past safe use (Participant 10's faulty hammer drill). The latter mechanism overlaps with the quality-control failures discussed in Theme 6 below, indicating that tool-related injuries in this sample stem from both individual behavior and organizational maintenance practices rather than a single root cause. These examples emphasize the importance of thorough training in tool use and regular maintenance checks to mitigate the risks of tool mishandling, highlighting a broader safety management issue that requires urgent attention.

#### 3. Health impacts of unsafe exposure.

Prolonged exposure to heat and cold, as well as hazardous substances including sewer gases, silica dust, and raw sewage, led to serious health problems reported by the participants. This is seen in Participant 4, who experienced smoke inhalation from a confined space. Participant 5 described chronic respiratory issues from repeated exposure to sewer gas while working on drains, indicating a gradual decline in health. In contrast, Participant 20 developed a staph infection after unprotected contact with sewage, highlighting the risk of sudden illness. Participant 1 experienced a health impact when the participant caught a piece of equipment, which later caused an intestinal blockage. Further, Participant 3 experienced a near-death situation involving heat exhaustion and a stroke. Lastly, Participant 21 explained that the absence of air movers increases the risk in confined spaces with sewage. These exposures follow two distinct risk profiles: acute incidents with a single identifiable point of exposure (Participant 20's staph infection, Participant 4's smoke inhalation) and cumulative exposure whose health effects emerged gradually over repeated contact (Participant 5's chronic respiratory decline from sewer gas). The two profiles call for different interventions—PPE and incident response for the former, ventilation standards and longitudinal health monitoring for the latter. These situations emphasize the need to implement stronger protective measures, such as improved ventilation, mandatory PPE, and health monitoring, to prevent both short- and long-term health damage.

#### 4. Challenges in training adoption.

Insufficient training, especially for new or subcontracted workers, was a significant barrier to adopting safe practices. Further, as seen in this study, systemic failures persist, such as inadequate training and poor equipment maintenance, when the perception gaps remain unaddressed. Participant 13 explained that Insufficient training on equipment such as scissor lifts and excavators was a problem. Participant 11 noted that there is insufficient safety training, especially for non-English speakers. Participant 2 noted that the subcontractor team received only a three-hour orientation instead of a full day and a half of training, which contributed to a near-fatal fall. Moreover, Participant 9 linked a grinder-related mistake to inadequate training in a hurried environment. This situation reveals a systemic issue in which brief, inadequate training, along with a rush to finish tasks, compromises safety. This further points to the need for more comprehensive, ongoing education tailored to the trade's specific needs. Participant 14 contends that insufficient training in safety or equipment operation creates risks. While Participant 18 understands the need for ongoing training to enhance safety awareness and prevent incidents. Across these accounts, the training gap was not evenly distributed: it clustered specifically around subcontracted crews (Participant 2), apprentices (Participant 13), and non-English-speaking workers (Participant 11), suggesting the deficit reflects unequal access to training within a company's workforce rather than a uniform shortfall in training quality overall.

#### 5. Lack of policies and monitoring.

Inconsistent safety policies and weak monitoring emerged as recurring issues that varied by company size. Smaller firms, as noted by Participant 3, often lacked formal reporting systems, leading to unrecorded incidents. Larger companies, according to Participant 20, sometimes overlook injuries without follow-up. This inconsistency highlights a significant gap in policy enforcement and accountability. This suggests a need for stricter regulations and regular safety evaluations to protect workers in the industry. Moreover, the lack of adherence to safety protocols suggests that the company is not monitoring or enforcing its policies. Participant 8 was working at heights on a roof alone without the aid of a coworker; this suggests a lack of policy for multiple workers when there are risks. Participant 12 acknowledges that enforcing glove use as a requirement for post-injury. Participant 13 acknowledges that failure to verify certifications for operating heavy equipment can present operational risks. The lack of policy is evident when Participant 17 sustains a dog bite and explains that there was no policy requiring customers to secure their pets. This gap appeared at both ends of the company size spectrum: smaller operations, such as Participant 3's, more often lacked a formal reporting system altogether, while larger operations, such as Participant 20's, had policies on paper that went unenforced after an incident. This suggests the core failure is inconsistent enforcement and follow-through rather than the absence of written policy alone.

#### 6. Absence of quality control.

Limited quality control over tools and equipment raised serious concerns, as defective or modified items increased the risk of injury. An example of quality control is Participant 16, who explained that a trailer detached due to improper securing prior to transport. Participant 10 described a fall from a duct-taped ladder, highlighting the dangers of makeshift repairs. Additionally, Participant 15 lost a finger in an incident involving a modified wire brush drill. This highlights the risks associated with unauthorized modifications. Further, Participant 7 tripped and fell into a hole where the grate had been removed, suggesting a lack of follow-up and quality control. Participant 22 underestimated the weight of sheetrock and sustained a back injury that a quality control check (weight approximated with a plan of action) could have prevented. This paradox of feeling safe despite inadequate protections mirrors the experiences of participants in this study. This absence of quality control operates differently from the tool-misuse incidents discussed in Theme 2 above: whereas those incidents centered on workers bypassing safety features under time pressure, the incidents here—the duct-taped ladder, the modified wire brush drill, the unsecured trailer—reflect equipment and site conditions that were never properly maintained or inspected in the first place, regardless of the worker's behavior in the moment. These incidents emphasize a broader lack of oversight into equipment maintenance. It further suggests that companies implement stricter quality standards and regular inspections to ensure workers’ safety and well-being.

#### 7. Lack of physical safety and insurance.

The absence of adequate physical safety structures and insurance support was a common issue, with incidents linked to unstable work conditions and insufficient financial protection. Meanwhile, employers, particularly in smaller firms, prioritize productivity over safety. This systemic silence perpetuates failures such as inadequate training and poor equipment maintenance. Participant 14 illustrated that hazardous environments, such as wet or dark areas with electrical hazards, contribute to incidents. Participant 18 fell through a plywood catwalk onto a bookshelf due to its poor structural integrity, illustrating a site hazard. Furthermore, Participant 18 again commented about a risky crawl space with water and exposed wiring. In contrast, Participant 20 received no compensation for an injury, reflecting limited insurance coverage in smaller firms. Participant 21 had a similar problem with a lack of company support for medical bills or paid time off after an on-the-job injury. Again, Participant 23 experienced an unsupportive employer with no paid time off for injury recovery, despite covering medical bills. These examples prove a need for improved physical safety features, such as reinforced structures, and comprehensive insurance to support injured workers, addressing both immediate and ongoing safety needs. Further exploration into Workers’ Compensation is also needed.

Owner and employee accounts diverged most clearly in terms of the cost and structure of safety. Among the six owner participants, several described running lean or solo operations that limited their capacity to invest in safety systems: Participant 5, who has operated his own plumbing franchise since 1988, explained that he does most work alone rather than hiring help he would need to redo—“I had a guy working for me a little, but I do it by myself”—while noting that going solo meant no longer maintaining a full fleet of trucks and heavy equipment. Participant 1, co-owner of his plumbing business with his spouse at the time of a serious lifting injury, noted that being the owner meant there was no one above him to report the incident to: “there was no one to report it to, me and the co-owner… I was never the one to complain about a job.” Participant 20, a business owner of six years, described safety investment as a competitive liability: “economic pressure as a business owner makes safety costly—shoring and PPE increase job costs, but customers prioritize low prices, favoring unsafe competitors.” Participant 12, a company CEO, described a more formalized response, noting that “every policy we have is from a mistake or mishap,” including a mandatory glove policy adopted only after an employee's injury. Two owner participants had shifted out of active ownership into oversight or training roles: Participant 8 ran his own business for several years before moving into a plumbing inspector role with a Texas city, and Participant 11 owned and operated plumbing businesses before shifting to safety training, recalling that dismissiveness toward injuries was once structural—“safety wasn't discussed when I started… if you got hurt, you bandaged it and kept working.” Participant 20 also recalled experiencing that same dismissiveness from the employee side earlier in his career: when he reported a work-related staph infection, “I informed my employer, who dismissed it, saying it could've happened anywhere.” Read together, these accounts suggest the perception gap described throughout this study is not simply employer indifference; ownership itself removes a layer of oversight and accountability—no supervisor to report to, competitive pressure that discourages investment in safety, or the capacity constraints of running a lean operation—even as several owners describe enforcing stricter policies within their own operations than they experienced, or created, as employees.

### 3.3 Incidents

This study identified 68 reported incidents which were categorized into 4 incident types (near misses = 7; physical injuries = 43; fatalities = 3; Occupational Health Risk = 15) from the interviews, which occurred in various workplace settings, including residential homes (e.g., crawlspaces, attics), commercial spaces (e.g., restaurants, convenience stores), industrial sites (e.g., hospitals, water treatment plants), and construction areas—many of which involved confined spaces or elevated structures. The tasks related to these incidents were diverse, including pipe cutting, soldering, sewer repair, drain clearing, water heater installation, and handling materials. Table 4 summarizes all the incidents and incident types.

**Table 4:** Participant Injury/near-miss.

Incident types	Incidents
Physical injuries (n = 43)	Bilateral inguinal hernias; cuts on arms from boiler threads; Knee tweak from a trench fall; finger injury from a pipe; skull fracture (witnessed); Knee tweak from a trench fall; finger injury from a pipe; Wrist injury from a fall; Knee tweak from a trench fall; finger injury from a pipe; helper ladder fall; Tripping into the floor drain; Hand injuries; Grinder hand injury; coworker wrist break; eye injury; nose injury; hand scars from pipes; hand injury from urinal porcelain; Ladder fall with hip contusion; Eye injuries from metal shards; attic stair collapse with bruise; sheetrock fall; finger laceration; Back injury from the I-beam; excavator bucket injury; Ceiling tile injury to helper; attic stair collapse injury; Elbow contusion; finger amputation; finger injury from a scissor lift; Pro-press finger injuries; hole saw finger skinning; dog bite; sheet metal head injury; Back injury; eye injury from shard; Sewer related infection (testicular); Broken leg from ladder fall; Rebar hand injury with staph; razor knife leg injury; sewer-related staph infection; retinal detachment from strap;
Near Misses (n = 7)	Ladder near-miss; Electrocution near-miss; near-drowning in a trench; sewer machine electrical near-miss; Chain failure with excavator tipping near-miss; trailer detachment; attic staircase collapse; Trench collapse with falling objects
Fatalities (n = 3)	Tommy lift fatality (witnessed); electrician electrocution (witnessed); Trench cave-in (fatality)
Occupational Health Risk (n = 15)	Heat exhaustion; excavator electrocution (heard from colleagues); Respiratory illness from job conditions; kidney stones; ethylene oxide exposure; sewer gas explosion; Smoke inhalation from soldering; dehydration with heat exhaustion; Inexperience with heat resulting in heat exhaustion and dehydration; Sewage exposure in the lift station; silica dust exposure; trapped under a house; Chronic respiratory issues from gases, dusts and chemicals; deviated septum; walking pneumonia from exposure

### 3.4 Accident types

The incidents included 43 personal injuries, 18 witnessed coworker injuries, and 7 near-misses, offering a clear view of safety risks. Personal injuries ranged from acute physical trauma like cuts (hand injury from a grinder), sprains (wrist sprain from tripping), and fractures (wrist injury from a drill) to chronic issues like respiratory problems from sewer gas exposure. Witnessed coworker injuries included severe cases, such as a near-fatal head injury from a mezzanine fall and a fatal trench cave-in. Near misses included critical events, such as electrocution risks and trench collapses, which demonstrated the potential for serious outcomes. The distribution of incidents across participants, with an average of approximately 3.0 incidents per person, reflected the widespread nature of safety issues. Table 5 presents examples of participants with types of tallied accidents.

**Table 5:** Accident type.

Accident Type	Count
Cuts, Lacerations, Abrasions	23
Musculoskeletal Injuries and/or Death	12
Confined Space/Environmental Near-Misses	5
Head/Facial Injuries	4
Respiratory/Chemical Exposure	4
Electrocution Hazards	4
Eye Injuries	3
Infections	3
Heat-Related Illnesses	3
Other Near-Misses	7

### 3.5 Contributing factors to incidents and accidents

Participant examples are summarized and identified as mostly routine operations, with some noting inexperience and novice ability. Most common, though, are typical daily workflow applications, including common tasks. These are summarized below and in Table 6, with participant examples and contributing factors to incidents and accidents.

**Table 6:** Contributing factors.

Contributing Factors (n = 39)
Inattention, rushing, fatigue, inexperience, complacency, improper tool use, and lack of vigilance; Lack of training, inadequate PPE, improper equipment, poor onboarding, lack of lockout/tagout, improper storage/installation; non-reporting, hustle culture, job pressure, fear of job loss, small business culture, historical attitudes, and loyal employee risk; Heavy lifting, ladder falls, tool injuries, electrocution, crushing; Wet ground, confined spaces, falling objects, electrical hazards, sewer gas; Respiratory illness, heat exhaustion, chemical exposure, kidney stones, silica dust; PPE adoption, protocol changes, safety advocacy, training improvements

- Tool and Equipment Use: Many cuts resulted from improper or rushed use of tools like press tools, grinders, razor knives, or wire brushes. A lack of training in tool safety exacerbated risks.
- Inattention and Rushing: Distraction or haste led to errors.
- PPE Underutilization: Failure to wear gloves or safety glasses increased injury severity.
- Environmental Hazards: Exposure to sharp materials in hazardous environments, like muddy rebar or sewer manholes, contributed to cuts and subsequent infections.
- Inexperience: Novice plumbers were more prone to tool-related cuts.

### 3.6 Recommendations from participants

These interviews reveal a complex interplay of environmental hazards, physical demands, human errors, inadequate resources, and a workplace culture that often prioritizes productivity over safety. While post-injury changes indicate some progress, they are typically reactive and highlight the need for proactive safety measures. Most participants made recommendations that reflected safety protocols. Table 7 summarizes these reflections alongside their implications for improving safety practices.

**Table 7:** Participant recommendations for improving safety.

Recommendation	Description	Participant Examples
Enhance PPE Use	Mandate gloves, safety glasses, face shields, hard hats, and respiratory protection to prevent cuts, eye injuries, and chemical exposure	1, 2, 3, 4, 5, 6, 8, 9, 11, 12, 15, 16, 17, 19, 20, 21, 22, 23
Improve Training	Provide regular training on tool use, confined spaces, electrical safety, and heat management to address inexperience and human errors	1, 2, 3, 4, 5, 6, 8, 9, 10, 11, 12, 13, 14, 15, 16, 17, 18, 20, 21, 22, 23
Manage Fatigue	Enforce rest schedules and limit overtime to reduce fatigue-related errors	2, 4, 8, 10, 15, 16, 18, 20, 22
Encourage Reporting	Create open reporting systems to counter “plumber mentality” and job security fears, ensuring prompt incident documentation	1, 2, 3, 7, 9, 10, 12, 13, 14, 15, 17, 19, 23
Strengthen Supervision	Increase oversight for inexperienced workers and verify equipment certifications	1, 2, 6, 7, 9, 10, 13, 14, 15, 16, 17, 18, 21, 22
Refuse Unsafe Tasks	Empower workers to prioritize safety and refuse hazardous tasks	4, 5, 11, 13, 14, 15, 18, 21, 23
Improve Equipment Safety	Ensure proper ladders, grounded tools, and lockout/tagout systems to prevent falls, electrical hazards, and equipment failures	2, 4, 6, 8, 10, 11, 12, 13, 14, 15, 16, 20
Address Environmental Hazards	Implement shoring, ventilation, and hazard assessments for trenches, confined spaces, and wet conditions	3, 4, 5, 6, 11, 12, 14, 15, 17, 18, 20, 21

## 4. Discussion

This qualitative study reveals persistent and multifaceted safety challenges in the Texas plumbing industry. Plumbers described co-occurring hazards—system failures, high-risk work practices, and adverse health effects—despite existing OSHA regulations and licensing requirements; while participants often described these factors as mutually reinforcing, the cross-sectional interview design cannot establish causal or dynamic relationships among them. The seven key themes identified—barriers to proper use of personal protective equipment (PPE), challenges in safe tool use, health impacts of unsafe exposure, challenges in training adoption, lack of policies and monitoring, absence of quality control, and lack of physical safety and insurance—mirror findings from previous research in high-risk trades [19,23–27] Because participants repeatedly described unsafe practices as behaviors learned from peers and supervisors, the cultural and behavioral themes below are interpreted through Bandura's [28] social cognitive theory, particularly observational learning and reciprocal determinism among behavior, environment, and personal cognition.

Consistent with prior studies in the construction sector, many plumbers reported injuries linked to inconsistent or improper use of PPE and tools. For example, participants described incidents such as wrist fractures from drill accidents without gloves and infections from exposure to sewage. These findings echo recent research documenting similar reluctance to use PPE due to discomfort or poor fit, time pressure, perceived low risk, inadequate training, and weak safety supervision [29]. Boakye et al. [30] identified key perceptual barriers among building construction artisans, including discomfort, inconvenience, and the belief that PPE interferes with task performance. Al-Bayati et al. [31] ranked poor risk perception, lack of safety training, inadequate supervision, and insufficient management support as the top contributors to PPE non-compliance using fuzzy-set analysis of construction sites. Seah et al. [32] further showed, via the COM-B model, that both physical capability (e.g., ergonomic discomfort and fit issues) and psychological capability (e.g., knowledge gaps about PPE benefits) significantly predict workers’ intention to wear PPE properly. The present study adds that even when PPE is available, practical barriers and workplace norms, particularly the pervasive “get-it-done” culture in Texas plumbing, often undermine its use. Experienced workers modeling unsafe shortcuts further normalizes non-compliance through observational learning.

Exposure to hazardous materials (e.g., sheetrock dust, sewage) and unsafe environments was a recurring theme. One participant noted, “I’ve inhaled sheetrock dust… realizing the long-term health risks.” Such accounts reinforce the need for improved hazard controls, as highlighted in Hyphen Solutions [33] and Lanjewar [1]. Organizational shortcomings, including inadequate training, lack of policy enforcement, and insufficient quality control, were frequently cited as contributing factors [34,35]. These issues are particularly acute in smaller firms, where formal reporting systems are often absent, leading to underreported incidents and missed opportunities for prevention [36].

A “get-it-done” culture, where speed is prioritized over safety, was commonly described. Consistent with social cognitive theory [28] and prior findings on why operatives engage in unsafe work behavior [37], experienced workers modeled unsafe behaviors that newer employees then reproduced through observational learning, normalizing shortcuts: “If they see an experienced guy skip safety steps… It’s permissive in their minds.” This aligns with findings from Lipscomb et al. [23] and underscores the importance of leadership and peer modeling—the reciprocal interplay of behavior, environment, and cognition central to social cognitive theory—in shaping safety practices, Lipscomb et al. [23]

The results highlight the urgent need for targeted interventions, including ongoing, practical training tailored to plumbers’ daily realities, improved access to ergonomic PPE, and standardized safety policies across companies of all sizes, consistent with diffusion-of-innovations principles for adopting new safety practices [38]. Supervisors and business owners should recognize production pressure and “hustle culture” as operational hazards and actively model safety-first behaviors. Treating safety as a core performance metric, rather than an interruption to productivity, could directly reduce injury and infection rates, lower insurance costs, and extend career longevity in the plumbing trade.

The perception gap between plumbers and employers, a theme that recurred across both owner (n = 6) and employee (n = 17) accounts in this sample, reflects issues in other high-risk jobs [20,39]. Plumbers often view safety measures as unrealistic, as demonstrated by Participant 9's injury sustained while rushing with a grinder. Meanwhile, employers, especially in small firms, prioritize productivity over safety. This disconnect results in systemic failures, such as inadequate training (e.g., Participant 2's subcontractor offering a brief orientation) and poor equipment maintenance (e.g., Participant 10’s duct-taped ladder). A collaborative approach, as suggested by Neubauer et al. [20], could help close this gap by involving plumbers, employers, and safety experts in co-designing solutions. For example, joint workshops could create tailored training that addresses plumbers’ practical concerns while meeting OSHA [19] standards.

The study's findings have broader significance for health in construction-related fields. The health effects of unsafe exposure, as seen in Participant 5's chronic respiratory issues from sewer gas and Participant 21's testicular infection from sewage, underscore the importance of long-term health monitoring. Furthermore, it suggests improved environmental controls, such as required ventilation in closed spaces. Similarly, the Lack of Physical Safety and Insurance, as evidenced by Participant 18's fall and Participant 20's unpaid injury, indicates an urgent need for stronger structural support and adequate insurance coverage (e.g., Workers’ Compensation). These issues align with NIOSH's [40] hierarchy of controls, which prioritizes engineering solutions (e.g., stronger catwalks) and administrative actions (e.g., mandatory personal protective equipment, or PPE) to reduce risks.

This paper contributes to industrial and management systems engineering by presenting a qualitative, worker-oriented viewpoint on safety challenges. It complements quantitative studies that typically focus on injury statistics [23]. The phenomenological approach reveals the real-life experiences behind the numbers. As seen in the emotional impact of unreported injuries or the dissatisfaction caused by inadequate training.

Once set up, this stage can measure the effects of suggested interventions, such as improved training or more vigorous policy enforcement. For example, future research could track changes in injury rates or worker perceptions after participatory safety programs. Further examples could be evaluating the cost-effectiveness of better PPE in small businesses.

## 5. Limitations

The study has limitations, including the low representation of women (only 1 female participant), which reflects the industry's male-dominated nature and may lead to the omission of gender-specific viewpoints. Additionally, coding was conducted by a single researcher without a second coder to calculate formal intercoder reliability; peer debriefing during theme refinement served as the primary check on interpretation, and future work would benefit from independent double-coding. Furthermore, relying on self-reported incidents may introduce recall bias. However, the study's focus on lived experiences prioritizes narrative depth over precise statistical accuracy. While the geographic diversity within Texas strengthens findings in that state, it may not fully reflect differences in other regions with distinct regulations or cultures. Member checking or respondent validation was not conducted; credibility was instead established through peer debriefing during theme refinement, and future studies could strengthen trustworthiness by returning summaries to participants for review. In summary, this study highlights closely interrelated system flaws, unsafe practices, and adverse health impacts in the Texas plumbing industry; given the cross-sectional design, these patterns are best understood as co-occurring rather than as evidence of a causally reinforcing cycle. This points to the need for a coordinated response.

Employment situations influenced the participants’ accounts. This impacted their safety practices and incident reporting. Self-employed plumbers, like Participant 3, often lacked formal reporting systems, resulting in underreported incidents, such as a knee injury sustained during a trench fall. Conversely, participants from large companies, such as Participant 12, described safety improvements following incidents, including enforcing the use of gloves after a coworker’s finger laceration. These differences showed that organizational factors play a significant role in safety outcomes. Including plumbers with direct experience of injuries or near-misses, the sample provided detailed insights into individual, environmental, and systemic factors. Thus, forming the basis for the seven themes identified in the analysis (e.g., Barriers to PPE Use, Challenges in Safe Tool Use).

## 6. Conclusion

This qualitative study provides a comprehensive analysis of the persistent safety and health challenges faced by plumbers in Texas. We identified seven themes: barriers to proper use of personal protective equipment (PPE), challenges in safe tool use, health impacts of unsafe exposure, difficulties in training adoption, lack of policies and monitoring, absence of quality control, and insufficient physical safety and insurance. These themes reflect the complex and systemic nature of occupational risks in the plumbing industry, as evidenced by incidents ranging from acute injuries (such as fractures and infections) to near-misses and chronic health conditions. Taken together, these findings point to structural gaps—training access, equipment maintenance, and consistent policy enforcement—operating alongside a workplace culture that pressures workers to bypass safety measures. Addressing both will require coordinated action from employers, licensing bodies, and researchers alike.

## Supporting information

S1 Table Participant-reported occupational injuries, illnesses, exposures, and near-miss incidents, categorized by work-system factors. (DOCX)
