# Supplementary material for: Occupational safety and health risks among plumbers: A qualitative study of workplace hazards, safety practices, and system-level gaps in Texas
Source: PLoS One. 2026 Aug 17;21(8):e0356512. doi: 10.1371/journal.pone.0356512 (PMC13480606; doi:10.1371/journal.pone.0356512)
Supplement: Supplementary file 1 [file pone.0356512.s001.docx]

**S1 Table. Participant-reported occupational injuries, illnesses, exposures, and near-miss incidents, categorized by work-system factors.**

| **_Participant ID_** | **_Injury_** | **_Body Part_** | **_Cause / Incident_** | **_Tools / Technology_** | **_Individual Factors_** | **_Organization_** | **_Tasks_** | **_Environment_** |
| --- | --- | --- | --- | --- | --- | --- | --- | --- |
| _Participant 1 (Incident 1)_ | _Bilateral inguinal hernias_ | _Abdomen / Groin_ | _Caught a dropped 1,100-pound boiler_ | _Manual lifting, no equipment_ | _Instinctive reaction, poor planning_ | _Limited staffing with trustees, no OSHA training_ | _Moving a heavy boiler onto the walkway_ | _County jail, confined space_ |
| _Participant 1 (Incident 1, secondary injury)_ | _Cuts_ | _Arms_ | _Threads on the boiler nipples during catch_ | _Boiler nipples (2.5-inch)_ | _Fatigue from prior work_ | _Self-owned business, no reporting_ | _Moving a heavy boiler_ | _County jail, confined space_ |
| _Participant 2 (Incident 2)_ | _Severe wrist sprain_ | _Right Hand_ | _Tripped over silt fence, braced fall with hand_ | _None_ | _Carelessness, distraction, and anger_ | _Lax reporting, safety concerns ignored_ | _Walking on a job site_ | _Cold morning, job site hazards_ |
| _Participant 2 (Incident 2)_ | _Head Injury (near fatal - witnessed)_ | _Head_ | _Tripped over a 2x4 frame opening in a mezzanine while walking backward, pulling wire, fell 9 ft, hard hat dislodged during fall._ | _Wire spool (electrician). No railing or barrier around mezzanine opening. The ladder is present but not relevant to the fall._ | _Lack of awareness (walking backward, unfamiliar with site), inadequate onboarding/training for new subcontractor crew (3-hour orientation vs. Standard day-and-a-half training)._ | _The prime contractor accepted responsibility for improper onboarding. The safety team failed to ensure proper training or install a railing. The job shut down for 2 weeks prior due to other injuries. Post-incident, safety culture improved._ | _An electrician is pulling wire through the mezzanine for control powering._ | _Mezzanine with 9-ft ceilings, featuring a 2x4 ft temporary frame opening for ladder access, with no railing or barrier. Job site near fire station (100 yards)._ |
| _Participant 2 (Incident 3)_ | _Stomach cut laceration from circular saw, non-fatal._ | _Stomach_ | _Carpenter on ladder, reaching 3 ft above head with circular saw (blade safety locked open), dropped saw after cutting 2x4, causing it to cut the stomach._ | _Circular saw with blade safety locked open._ | _Unsafe behavior (such as standing backward on a ladder, reaching excessively, or disabling safety mechanisms) was perceived as “stupid” by the crew, with no cultural shift prompted._ | _Strict safety rules (gloves, safety glasses, hard hats, tie-offs). No specific organizational failure_ | _Cutting a 2x4 on a ladder (individual error)_ | _Construction site with strict safety protocols, no specific environmental hazards noted._ |
| _Participant 2 (Incident 4)_ | _Thumb injury: nearly severed by hacksaw_ | _Thumb_ | _Electrician cutting conduit with hacksaw, thumb positioned incorrectly (pointed down pipe instead of wrapped around), saw jumped, cutting thumb._ | _Hacksaw, conduit pipe._ | _Improper technique (using thumb to mark cut without moving it), perceived as individual error (“not a smart guy”), no cultural shift prompted._ | _Job site with strict safety rules. No specific organizational failure was noted beyond individual error._ | _Cutting conduit pipe._ | _Construction site with strict safety protocols, no specific environmental hazards noted._ |
| _Participant 3 (Incident 1)_ | _Knee tweak_ | _Knee_ | _Fell into the trench due to the wet ground giving way_ | _Work boots_ | _Fatigue, lack of awareness_ | _Self-owned business, no reporting_ | _Preparing for inspection_ | _Wet residential ground_ |
| _Participant 3 (Incident 2)_ | _Finger injury_ | _Left Hand_ | _Pulled 4-inch PVC pipe with 3-inch pipe inside_ | _PVC pipes, no gloves_ | _Haste, poor judgment_ | _Self-owned business, no reporting_ | _Pulling pipes from the rack_ | _Shop storage area_ |
| _Participant 4 (Incident 1)_ | _Smoke inhalation_ | _Lungs_ | _Inhaled gas/smoke in a confined space without ventilation_ | _Soldering torch, no blower_ | _Naivety, job priority over health_ | _Large company, later safety focus_ | _Soldering copper pipes_ | _Under the beam house, confined_ |
| _Participant 4 (Incident 2)_ | _Witnessed electrocution_ | _Body (coworker)_ | _The electrician bumped into the live panel box_ | _Electrical panel_ | _Distraction, inexperience_ | _Large company, safety meetings_ | _Remodeling restaurant_ | _Underground, job site_ |
| _Participant 4 (Incident 3)_ | _Witnessed a ladder fall_ | _Body (coworker)_ | _The helper fell from a ladder onto a plastic floor_ | _8-ft ladder, no bracing_ | _Lack of training, haste_ | _Large company, later safety focus_ | _Inspecting a water leak_ | _Residential, plastic-covered_ |
| _Participant 5 (Incident 1)_ | _Respiratory illness_ | _Respiratory tract_ | _Chronic exposure to sewer gases_ | _Sewer tools, no PPE_ | _Long-term exposure, ignorance_ | _Self-owned, later avoided risks_ | _Drain work, sewer repair_ | _Filthy houses, sewer-filled_ |
| _Participant 5 (Incident 2)_ | _Chemical exposure_ | _Face/Lungs_ | _Ethylene oxide discharge from an uncharged valve_ | _Valve, no PPE_ | _Ignorance, employer negligence_ | _Contractor, no compensation_ | _Valve replacement_ | _Hospital, indoor_ |
| _Participant 5 (Incident 3)_ | _Explosion near-miss_ | _Body_ | _Torch ignited gas with blue glue_ | _Torch, blue glue_ | _Inattention, lack of protocol_ | _Self-owned, no formal training_ | _Sweat joint installation_ | _Restaurant, ditch_ |
| _Participant 6 (Incident 1)_ | _Sewage exposure_ | _Body_ | _Soaked in sewage when the lift station is activated_ | _Pumps, no PPE_ | _Youth, lack of forethought_ | _Lax safety culture, no change_ | _Pump repair_ | _Lift station, confined_ |
| _Participant 6 (Incident 2)_ | _Witnessed a crushing death_ | _Body (coworker)_ | _Trapped by tommy lift on the truck bed_ | _Tommy lift_ | _Inexperience, distraction_ | _Inspection role, OSHA involved_ | _Material unloading_ | _Commercial job site_ |
| _Participant 7_ | _Minor fall injury_ | _Body_ | _Tripped into an open floor drain during inspection_ | _None_ | _Lack of situational awareness_ | _Supportive, documented_ | _Sprinkler system inspection_ | _Construction site_ |
| _Participant 8 (Incident 1)_ | _Hand injuries (scars)_ | _Hands_ | _Cuts and scrapes from pipes and tools without gloves_ | _Pipes, tools_ | _Dexterity needs, time pressure_ | _Self-owned, later inspection_ | _General plumbing tasks_ | _Various job sites_ |
| _Participant 8 (Incident 2)_ | _Ladder near-miss_ | _Arms/Body_ | _The ladder slipped on the sloped driveway_ | _Short ladder_ | _Solo work, poor setup_ | _Self-owned, minimal training_ | _Clearing a clogged drain_ | _Residential, sloped driveway_ |
| _Participant 8 (Incident 3)_ | _Heat exhaustion_ | _Body_ | _Dehydration from intense heat work_ | _None_ | _Youth, lack of hydration_ | _Hustle culture, no treatment_ | _General plumbing tasks_ | _Hot regional climate_ |
| _Participant 9 (Incident 1)_ | _Grinder injury_ | _Hand_ | _The grinder slipped without a guard_ | _Angle grinder, no guard_ | _Lack of training, haste_ | _Small company, no reporting_ | _Cutting cast iron_ | _Condo, indoor_ |
| _Participant 9 (Incident 2)_ | _Coworker wrist injury_ | _Wrist (coworker)_ | _Drill jammed, spun, and broke the wrist_ | _Drill, no clutch_ | _Inexperience, poor bracing_ | _Small company, no reporting_ | _Drilling wood_ | _Another state, job site_ |
| _Participant 9 (Incident 3)_ | _Eye injury (coworker)_ | _Eye/Face (coworker)_ | _Metal shard in the eye from grinding without glasses_ | _Grinder, no safety glasses_ | _Ignoring advice, inexperience_ | _Small company, no formal action_ | _Grinding cast iron_ | _Tunnel, confined_ |
| _Participant 9 (Incident 4)_ | _Nose injury_ | _Nose_ | _The pipe slipped while cutting in the crawl space_ | _Bandsaw_ | _Inattention, confined space_ | _Current company, reported_ | _Cutting galvanized pipe_ | _Crawl space, low clearance_ |
| _Participant 9 (Incident 5)_ | _Arm gash_ | _Arm_ | _The hot sawzall blade rested on the arm_ | _Sawzall_ | _Stupidity, rough life phase_ | _Small company, no reporting_ | _Cutting wood_ | _Job site, unspecified_ |
| _Participant 9 (Incident 6)_ | _Heat exhaustion_ | _Body_ | _Worked in a hot attic without breaks_ | _None_ | _Lack of hydration, inattention_ | _Small company, self-managed_ | _Attic work_ | _Hot Texas attic_ |
| _Participant 10 (Incident 1)_ | _Hip contusion_ | _Hip_ | _Fell from a faulty ladder, hitting a concrete wall_ | _Faulty ladder (duct-taped)_ | _Inexperience, borrowed equipment_ | _Supportive, followed up_ | _Soldering copper pipes_ | _Business basement, confined_ |
| _Participant 10 (Incident 2)_ | _Heat exhaustion_ | _Body_ | _Dizziness from dehydration in a hot basement_ | _None_ | _Youth, job focus over health_ | _Supportive, informal reminder_ | _Gluing fittings_ | _Hot basement, indoor_ |
| _Participant 11 (Incident 1)_ | _Electric shock near-miss_ | _Body_ | _Shocked while wiring a pump near a live outlet_ | _110V outlet, no lockout_ | _Complacency, no PPE_ | _Self-owned, no formal action_ | _Pump installation_ | _Convenience store, hot day_ |
| _Participant 11 (Incident 2)_ | _Near-drowning near-miss_ | _Body (worker)_ | _The trench flooded due to valve activation_ | _Trench boxes, no lockout_ | _Lack of protocol, operator error_ | _Large company, safety shift_ | _Water line repair_ | _Water treatment plant, deep_ |
| _Participant 12_  _(Incident 1)_ | _Eye injuries (coworker)_ | _Eye/Face (coworker)_ | _Metal shards in the eyes from sawing/threading pipes_ | _Saw, threading tools_ | _Sweat, prior eye issues_ | _Large company, policy change_ | _Pipe work_ | _Various job sites_ |
| _Participant 12 (Incident 2)_ | _Stomach bruising (coworker)_ | _Torso/Body (coworker)_ | _Fell from the collapsing attic stairs_ | _Attic stairs (finishing nails)_ | _Inexperience, poor installation_ | _Large company, safety focus_ | _Water heater installation_ | _Residential attic_ |
| _Participant 12 (Incident 3)_ | _Body bruising (coworker)_ | _Body (coworker)_ | _Fell through the sheetrock, hitting the joist_ | _Attic - joists_ | _Inexperience, delayed reporting_ | _Large company, safety focus_ | _General attic work_ | _Residential attic_ |
| _Participant 12 (Incident 4)_ | _Finger laceration (coworker)_ | _N/A (coworker)_ | _Cut from the water heater jacket without gloves_ | _Water heater_ | _Refusal to use PPE_ | _Large company, enforced gloves_ | _Carrying a water heater_ | _Job site, unspecified_ |
| _Participant 13 (Incident 1)_ | _Back injury_ | _Back_ | _The I-beam fell during the use of a scissor lift use_ | _Scissor lift, I-beam_ | _Inexperience, lack of training_ | _No support, high turnover_ | _Removing I-beams_ | _Hospital interstitial space_ |
| _Participant 13 (Incident 2)_ | _Chest bruise (coworker)_ | _Torso/Body (coworker)_ | _Hit by an excavator bucket in the swing zone_ | _Excavator_ | _Inexperience, operator error_ | _No certification checks_ | _Spotting for an excavator_ | _Rocky terrain, job site_ |
| _Participant 14 (Incident 1)_ | _Facial injury (helper)_ | _Face_ | _Hit by a falling plywood ceiling tile_ | _Plywood tile, ladder_ | _Inattention, wet conditions_ | _Lax response, minimal action_ | _Fixing a water leak_ | _Dark warehouse, wet_ |
| _Participant 14 (Incident 2)_ | _Near-electrocution_ | _Body_ | _Sewer machine sparked due to an ungrounded outlet_ | _Sewer machine, no tester_ | _Lack of initial check_ | _Supportive, protocol added_ | _Clearing mainline stoppage_ | _Job site, unspecified_ |
| _Participant 14 (Incident 3)_ | _Near-fall (helper)_ | _Body (helper)_ | _The attic staircase collapsed under the weight_ | _Attic staircase_ | _Poor installation, weight excess_ | _Supportive, policy change_ | _Water heater replacement_ | _Older house, attic_ |
| _Participant 15 (Incident 1)_ | _Elbow contusion_ | _Elbow_ | _Slipped while using the snap ratchet cutter in a tight space_ | _Snap ratchet cutter_ | _Inattention, tight space_ | _Large company, followed protocol_ | _Pipe cutting_ | _Elevated structure, ceiling_ |
| _Participant 15 (Incident 2)_ | _Finger amputation_ | _Finger_ | _Glove caught in wire brush drill_ | _Wire brush drill_ | _Tool modification, inattention_ | _Large company, safety focus_ | _Cleaning copper fittings_ | _Job site, unspecified_ |
| _Participant 15 (Incident 3)_ | _Finger injury_ | _Finger_ | _Hit the all-thread on the scissor lift_ | _Scissor lift, trapeze hanger_ | _Lack of awareness_ | _Large company, safety focus_ | _Retrieving tool_ | _16-story job site_ |
| _Participant 15 (Incident 4)_ | _Silica dust exposure_ | _Lungs_ | _Inhaled dust while hammer drilling without a mask_ | _Hammer drill, no mask_ | _Ignorance, long exposure_ | _Large company, later mandated_ | _Concrete floor drilling_ | _Indoor, job site_ |
| _Participant 16 (Incident 1)_ | _Excavator chain near-miss_ | _Body_ | _The chain came loose, dropping material_ | _Excavator, chains_ | _Lack of training, chance positioning_ | _Large company, blame shifted_ | _Moving material_ | _Job site, unspecified_ |
| _Participant 16 (Incident 2)_ | _Excavator tipping near-miss_ | _Body_ | _Mini excavator tipped due to improper weight distribution_ | _Mini excavator_ | _Lack of training, inexperience_ | _Large company, training later_ | _Moving material_ | _Job site, unspecified_ |
| _Participant 16 (Incident 3)_ | _Trailer detachment near-miss_ | _Body_ | _The trailer detached due to improper securing_ | _Trailer_ | _Inadequate training, no double-check_ | _Large company, policy added_ | _Preparing trailer_ | _Shop yard_ |
| _Participant 16 (Incident 4)_ | _Sheetrock dust exposure_ | _Lungs_ | _Inhaled dust during cutting causes headaches_ | _None_ | _Ignorance, no mask_ | _Large company, later OSHA training_ | _Cutting sheetrock_ | _Indoor, job site_ |
| _Participant 17 (Incident 1)_ | _Dog bite_ | _Buttocks_ | _Bit by a dog after being waved into the house_ | _None_ | _Lack of caution, customer error_ | _Supportive, reported, but informal_ | _Entering house_ | _Residential, indoor_ |
| _Participant 17 (Incident 2)_ | _Finger injuries (coworkers)_ | _Fingers/hand (coworkers)_ | _The press tool crushed fingers during crimping_ | _Pro press tool_ | _Inattention, rushing_ | _Small company, unreported_ | _Prefabbing connections, pipe work_ | _Outside the van, the house_ |
| _Participant 17 (Incident 3)_ | _Finger skinning (coworker)_ | _Finger (coworker)_ | _Hole saw on drill triggered, skinning finger_ | _Hole saw, drill_ | _Rushing, no gloves_ | _Small company, unreported_ | _Tightening the hole saw_ | _Job site, unspecified_ |
| _Participant 17 (Incident 4)_ | _Trapped under the house (coworker)_ | _Trunk (coworker)_ | _Fell into a hole in the crawlspace, legs numb_ | _None_ | _Inattention, poor supervision_ | _Small company, no training_ | _Working in the crawlspace_ | _Residential, crawlspace_ |
| _Participant 17 (Incident 5)_ | _Head injury (coworker)_ | _Head (coworker)_ | _Hit by a falling sheet of metal_ | _Sheet metal_ | _Improper securing_ | _Small company, no details_ | _Unspecified task_ | _Job site, unspecified_ |
| _Participant 18 (Incident 1)_ | _Back injury_ | _Lower Back_ | _My back gave out carrying a 20-ft copper pipe_ | _Copper pipe_ | _Fatigue, improper movement_ | _Supportive medical support_ | _Carrying pipe_ | _Warehouse_ |
| _Participant 18 (Incident 2)_ | _Ditch collapse near-miss_ | _Body_ | _The trench collapsed due to wet, heavy soil_ | _Excavator, no trench box_ | _Inexperience, soil misjudgment_ | _Supportive, safety coordinator_ | _Prepping ditch_ | _Job site, wet conditions_ |
| _Participant 18 (Incident 3)_ | _Catwalk fall (coworker)_ | _Body (coworker)_ | _Fell through the plywood catwalk onto the bookshelf_ | _Plywood catwalk, all-thread_ | _Structural failure, inexperience_ | _Supportive, reported_ | _Working above the library_ | _School library, elevated_ |
| _Participant 18 (Incident 4)_ | _Vehicle accident near-miss_ | _Body_ | _T-boned in a van on an icy road during an emergency call_ | _Van_ | _Weather, fatigue_ | _Unsupportive manager, repairs_ | _Driving to the job_ | _Icy road, northern region_ |
| _Participant 18 (Incident 5)_ | _Electrocution risk near-miss_ | _Body_ | _Refused entry to the crawlspace with water and wiring_ | _Pump_ | _Safety awareness_ | _Retaliation for refusal_ | _Assessing crawlspace_ | _Residential, standing water_ |
| _Participant 19_ | _Eye injury (retinal detachment)_ | _Left Eye_ | _The strap snapped back while securing the equipment_ | _Strap_ | _Distraction by the customer_ | _Supportive systems, unreported_ | _Securing equipment_ | _Plumbing van_ |
| _Participant 20 (Incident 1)_ | _Staph infection_ | _Forearm_ | _Exposure to a sewer manhole during pipe tie-in_ | _None_ | _Lack of PPE, sewer exposure_ | _Dismissive, no support_ | _Drilling for sewer tie-in_ | _Sewer manhole_ |
| _Participant 20 (Incident 2)_ | _Walking pneumonia_ | _Lungs_ | _Overworking leads to 90% lung blockage_ | _None_ | _Fatigue, employer pressure_ | _Reluctant time off_ | _General plumbing tasks_ | _Various job sites_ |
| _Participant 20 (Incident 3)_ | _Hand injury (coworker)_ | _Hand (coworker)_ | _Cut by urinal pieces thrown into the dumpster_ | _Urinal, dumpster_ | _Haste, improper disposal_ | _Safety coordinator, investigated_ | _Disposing of the urinal_ | _Job site, dumpster_ |
| _Participant 20 (Incident 4)_ | _Fatal trench cave-in (coworker)_ | _Body (coworker)_ | _The trench collapsed without shoring_ | _None_ | _Lack of shoring, inexperience_ | _News-reported, no internal details_ | _Unspecified task_ | _Trench, job site_ |
| _Participant 21_ | _Infection_ | _Testicle_ | _Sewage exposure through a low-quality Tyvek suit_ | _Tyvek suit, no air movers_ | _Helper error, inadequate PPE_ | _Unsupportive, no compensation_ | _Clearing a cast iron pipe_ | _Tunnel, sewage-filled_ |
| _Participant 22 (Incident 1)_ | _Twisted ankle_ | _Ankle_ | _Sheetrock pile fell during counting_ | _Sheetrock_ | _Inexperience, underestimating weight_ | _Supportive, HR reported_ | _Counting sheetrock_ | _Job site, unspecified_ |
| _Participant 22 (Incident 2)_ | _Head injury (coworker)_ | _Head (coworker)_ | _Struck by a falling flue pipe, no hard hat_ | _Flue pipe_ | _No PPE, loose collar_ | _Safety-conscious, ambulance_ | _Boiler work_ | _Job site, unspecified_ |
| _Participant 22 (Incident 3)_ | _Fall from ladder (coworker)_ | _Body (coworker)_ | _Fell 30 ft from ladder, improper harness_ | _Ladder, no proper harness_ | _Inexperience, improper equipment_ | _OSHA fined, safety increased_ | _Carrying tools_ | _Elevated job site_ |
| _Participant 23 (Incident 1)_ | _Rebar hand injury with staph_ | _Right Hand_ | _Impaled hand on rebar while digging_ | _Sawzall, shovel_ | _Haste, no assistance_ | _Lax culture, medical coverage_ | _Fixing a water leak_ | _Pool deck, muddy hole_ |
| _Participant 23 (Incident 2)_ | _Razor knife leg injury_ | _Leg_ | _The knife slipped while trimming CPVC on the leg_ | _Razor knife, CPVC pipe_ | _Haste, improper positioning_ | _Prompt medical support_ | _Replacing waste/overflow_ | _Under the bathtub, slab_ |
