# Supplementary material for: Occupational safety and health risks among plumbers: A qualitative study of workplace hazards, safety practices, and system-level gaps in Texas
Source: PLoS One. 2026 Aug 17;21(8):e0356512. doi: 10.1371/journal.pone.0356512 (PMC13480606; doi:10.1371/journal.pone.0356512)
Supplement: Supplementary file 2 [file pone.0356512.s003.docx]

RE: Response to Reviewers: *“Occupational Safety and Health Risks Among Plumbers: A Qualitative Study of Workplace Hazards, Safety Practices, and System-Level Gaps in Texas”*

Dear Editor and Reviewers,

We thank the Academic Editor and all three reviewers for their careful and constructive engagement with our manuscript. We are grateful for such a thorough reading of our manuscript. Their comments prompted a substantial revision: we reframed our methodological approach, added cross-participant analytic synthesis throughout the Results, grounded our discussion of the owner/employee perception gap in the study's own sample, and corrected a number of reporting and citation errors. We address each point below, with section numbers referring to the revised submission.

**Reviewer #1**

1. *Husserlian phenomenology vs. Braun & Clarke thematic analysis.* The reviewer is correct that the original framing did not match the analysis performed. The study is now described throughout as a qualitative descriptive design using Braun and Clarke's (2006, 2019) reflexive thematic analysis; the Husserlian/transcendental phenomenology language has been removed from §2.3, along with the now-uncited Husserl (1970) reference. The researcher's reflexive bracketing practice was retained, as it fits comfortably within reflexive thematic analysis.
2. *Data saturation not operationalized.* Agreed. §2.5 now states the stopping rule that was actually used: new codes were tracked per interview, and data collection continued until several consecutive transcripts introduced nothing new.
3. *"Self-reinforcing cycle" and "joint effort" overstate what a cross-sectional design can show.* We have softened this language in the Abstract, Discussion, and Limitations, describing the problems documented as co-occurring rather than causally reinforcing.
4. *Precision of "68 incidents" and "2.956 incidents per person"; missing intercoder reliability.* The per-person figure is rounded to approximately 3.0. We have also added a direct disclosure that coding was conducted by a single researcher, with the research team's Zoom discussions functioning as peer debriefing rather than independent double-coding, and that no formal reliability statistic was calculated (§2.7 and §6).
5. *Table 4 category overlap (e.g., staph infection under physical injuries vs. walking pneumonia under occupational health risk).* This was a fair point. We applied a consistent classification rule going forward: incidents originating from a discrete mechanical or traumatic event are physical injuries; illnesses or infections arising primarily from exposure are occupational health risks. The sewer-related staph infection and testicular infection have been moved into Occupational Health Risk under this rule, with counts recalculated (Physical injuries n=41; Occupational Health Risk n=17; total unchanged at 68).
6. *Percentages that don't sum correctly; spurious decimal precision.* Table 2 percentages are now rounded to one decimal. We also split the former "Employment Type" variable into two independent groupings, Business Structure (self-employed/small company/large company) and Role (owner/employee), with a footnote noting the two can overlap for a given participant.
7. *Grammar, table mislabeling, citation formatting.* "Participants comprised of" has been corrected to "Participants comprised"; the interview guide and participant-characteristics tables are now numbered and cross-referenced consistently as Table 1 and Table 2; and the broken chrome-extension:// URL in the Yi (2023) reference now points to a standard URL.
8. *Interviewer's dual role and an unexcerpted reflexive journal.* We appreciate the push for transparency here. §2.6 now includes three direct excerpts from the researcher's reflexive journal: two showing moments where OSHA/plumbing expertise prompted an urge to probe further that the researcher consciously held back, and a third showing restraint driven by sensitivity to a participant's injury rather than professional judgment.
9. *Results read as participant-by-participant cataloging rather than analytic argument.* We restructured §3.2 to add cross-participant synthesis to all seven themes — for instance, noting that PPE noncompliance spans experience levels rather than concentrating among newer workers, distinguishing behavioral from maintenance-driven tool-use incidents, separating acute from cumulative health-exposure profiles, and showing that policy-enforcement gaps appear at both ends of company size.
10. *Thin theoretical framing.* We have taken up Bandura's (1986) social cognitive theory as an organizing lens for the cultural and behavioral themes in the Discussion, applying it directly to the "get-it-done" culture findings (observational learning, reciprocal determinism) rather than leaving it as an uncontextualized citation.
11. *"There are no studies that discuss the topics at hand" is too sweeping.* Revised to state that plumber-specific qualitative research remains limited relative to the broader construction safety literature. We also identified that Osburn, Lee, and Gambatese (2022) was cited in-text but missing from the reference list; it has been added.
12. *Table 4's three fatalities — witnessed, experienced, or secondhand?* Clarified: two are labeled witnessed directly, and the trench cave-in is labeled reported secondhand, from a different company than the participant's own.
13. *Owner/employee split (6 owners, 17 employees) not systematically compared.* §3.2 now includes a comparative analysis across all six owner participants, examining how ownership itself removes a layer of oversight and accountability (no supervisor to report to, competitive cost pressure, the capacity limits of a lean operation), even as several owners describe enforcing stricter policies than they experienced as employees.
14. *No mention of member checking.* Correct — none was conducted. §6 now states this plainly, noting that peer debriefing during theme refinement served as the primary credibility check.
15. *Table 6 promises frequencies it doesn't provide.* The lead-in sentence has been corrected to remove that claim.
16. *Avetta (2025) and NIOSH (2018) citation consistency.* Avetta (2025) is now cited in-text alongside Neubauer et al. (2019) in the perception-gap discussion. NIOSH (2018) was already cited correctly and matches the reference list.

**Reviewer #2**

1. *Abstract lacks a stated gap, blends objective/methods/results, and overstates its conclusion.* The Abstract now states the research gap before the aim, separates Objectives/Methods/Results/Conclusion more cleanly, names the most analytically central themes rather than listing all seven with equal weight, and tempers the concluding claim to the scope a 23-participant qualitative sample can support.
2. *Introduction's absolute claim and thin differentiation from general construction literature.* The overbroad "no studies" claim has been replaced with a more precise statement of the gap; we have also strengthened our use of plumbing-specific comparators in the Discussion (see next point).
3. *Suggested Ghana IJBPA article (Danso et al., 2025).* Added to the reference list and cited in-text in the Discussion, where its institutional and compliance theory findings help explain why weak enforcement, rather than the absence of standards, appears to drive plumbing-safety noncompliance.
4. *Inconsistent consent reporting (electronic written vs. verbal).* Reconciled — the manuscript now describes verbal consent consistently throughout.
5. *Descriptive phenomenology paired with Braun & Clarke thematic analysis, unjustified.* Addressed as described under Reviewer #1, point 1: the methodological label now matches the analysis performed.
6. *Recruitment, saturation, and trustworthiness underexplained.* §2.4–2.5 now describe recruitment as drawing on a dataset of licensed Texas plumbers who had completed continuing education courses, contacted via a distributed recruitment statement. Saturation tracking is operationalized as described under Reviewer #1, point 2, and the absence of member checking is now stated directly rather than left silent.
7. *Participant table mislabeled; injury types not analytically distinguished; frequencies unexplained.* Table numbering is corrected throughout; the physical-injury/occupational-health-risk boundary is clarified and reapplied as described under Reviewer #1, point 5; and the Table 6 frequency claim has been corrected.
8. *Discussion leans on general literature over the study's own data; perception gap not tied to owner/employee data.* We now distinguish more clearly between claims grounded in participant accounts and claims drawn from the wider literature, bring in Danso et al. (2025) and Mbada et al. (2022) as plumbing-specific comparators, and ground the perception-gap discussion directly in the owner-versus-employee comparison described above.

**Reviewer #3**

1. *Abstract's Objectives section opens with methodology, not the aim.* Corrected — the Objectives sentence now states the research aim and gap without embedding method-level detail.
2. *Table numbering error (line 179).* Corrected; Table 1 (Interview Guide) and Table 2 (Participant Characteristics) are now numbered and referenced consistently.
3. Comments 3–4: How participants were selected and how contact information was obtained. §2.4 now states that recruitment drew on a dataset of licensed Texas plumbers who had completed continuing education courses, with a recruitment statement distributed to that group.
4. *Comment 5: Interview guide lacks an anonymity/confidentiality statement.* Added as the opening item of the interview guide (Table 1).
5. *Comment 6: Interview guide seems too brief to generate the range of Results reported.* The employee and owner question sets differ in depth by design — the employee protocol was built for detailed incident-level narrative, since employees perform the frontline tasks under study, while the owner protocol focused on management-level oversight. The semi-structured format allowed organic follow-up beyond the printed items, which accounts for the richer detail reported in Results.
6. *Comment 7: How was Table 2's information obtained, given the guide doesn't ask for it directly?* §3.1 now states that years of experience and employment classification were elicited through organic follow-up during the interview rather than the fixed guide questions.
7. *Comment 8: Table 7 caption incomplete ("Factors" of what?).* Corrected to "Table 7: Participant Recommendations for Improving Safety," matching §3.6.
8. *Comment 9: Study recommendations must be provided.* A new researcher-authored Recommendations section (§5) has been added, distinct from the participant-reported recommendations in Table 7, addressing training access, equipment-maintenance protocols, consistent policy enforcement, joint owner-employee safety planning, and insurance or bidding incentives for documented safety investment.

We thank the reviewers again for pushing us toward a more rigorous and transparent manuscript.

Grateful,

Mary Conger, MS, and Avishek Choudhury, PhD
