# Supplementary material for: Occupational safety and health risks among plumbers: A qualitative study of workplace hazards, safety practices, and system-level gaps in Texas
Source: PLoS One. 2026 Aug 17;21(8):e0356512. doi: 10.1371/journal.pone.0356512 (PMC13480606; doi:10.1371/journal.pone.0356512)
Supplement: Peer review history

# Peer review: Decision Letter 0

**DOI:** 10.1371/journal.pone.0356512.r001

**Authors:** Bismark Dwumfour-Asare

8 Jun 2026

Dear Dr. Choudhury,

Thank you for submitting your manuscript to PLOS ONE. After careful consideration, we feel that it has merit but does not fully meet PLOS ONE’s publication criteria as it currently stands. Therefore, we invite you to submit a revised version of the manuscript that addresses the points raised during the review process.

==============================

==============================

Please submit your revised manuscript by Jul 23 2026 11:59PM. If you will need more time than this to complete your revisions, please reply to this message or contact the journal office at plosone@plos.org. When you're ready to submit your revision, log on to https://www.editorialmanager.com/pone/ and select the 'Submissions Needing Revision' folder to locate your manuscript file.

- A letter that responds to each point raised by the academic editor and reviewer(s). You should upload this letter as a separate file labeled 'Response to Reviewers'.
- A marked-up copy of your manuscript that highlights changes made to the original version. You should upload this as a separate file labeled 'Revised Manuscript with Track Changes'.
- An unmarked version of your revised paper without tracked changes. You should upload this as a separate file labeled 'Manuscript'.

If you would like to make changes to your financial disclosure, please include your updated statement in your cover letter. Guidelines for resubmitting your figure files are available below the reviewer comments at the end of this letter.

If applicable, we recommend that you deposit your laboratory protocols in protocols.io to enhance the reproducibility of your results. Protocols.io assigns your protocol its own identifier (DOI) so that it can be cited independently in the future. For instructions see: https://journals.plos.org/plosone/s/submission-guidelines#loc-laboratory-protocols. Additionally, PLOS ONE offers an option for publishing peer-reviewed Lab Protocol articles, which describe protocols hosted on protocols.io. Read more information on sharing protocols at https://plos.org/protocols?utm_medium=editorial-email&utm_source=authorletters&utm_campaign=protocols.

As the corresponding author, your ORCID iD is verified in the submission system and will appear in the published article. PLOS supports the use of ORCID, and we encourage all coauthors to register for an ORCID iD and use it as well. Please encourage your coauthors to verify their ORCID iD within the submission system before final acceptance, as unverified ORCID iDs will not appear in the published article. *Only*  the individual author can complete the verification step; PLOS staff *cannot*  verify ORCID iDs on behalf of authors.

We look forward to receiving your revised manuscript.

Kind regards,

Bismark Dwumfour-Asare, MSc, PhD

Academic Editor

PLOS One

Journal Requirements:

When submitting your revision, we need you to address these additional requirements.

1. Please ensure that your manuscript meets PLOS ONE's style requirements, including those for file naming. The PLOS ONE style templates can be found at

https://journals.plos.org/plosone/s/file?id=wjVg/PLOSOne_formatting_sample_main_body.pdf and

https://journals.plos.org/plosone/s/file?id=ba62/PLOSOne_formatting_sample_title_authors_affiliations.pdf.

2. We note that your Data Availability Statement is currently as follows: [All relevant data supporting the findings of this study are included within the manuscript.  ]

Please confirm at this time whether or not your submission contains all raw data required to replicate the results of your study. Authors must share the “minimal data set” for their submission. PLOS defines the minimal data set to consist of the data required to replicate all study findings reported in the article, as well as related metadata and methods (https://journals.plos.org/plosone/s/data-availability#loc-minimal-data-set-definition).

For example, authors should submit the following data:

- The values behind the means, standard deviations and other measures reported;

- The values used to build graphs;

- The points extracted from images for analysis.

Authors do not need to submit their entire data set if only a portion of the data was used in the reported study.

If your submission does not contain these data, please either upload them as Supporting Information files or deposit them to a stable, public repository and provide us with the relevant URLs, DOIs, or accession numbers. For a list of recommended repositories, please see https://journals.plos.org/plosone/s/recommended-repositories.

If there are ethical or legal restrictions on sharing a de-identified data set, please explain them in detail (e.g., data contain potentially sensitive information, data are owned by a third-party organization, etc.) and who has imposed them (e.g., an ethics committee). Please also provide contact information for a data access committee, ethics committee, or other institutional body to which data requests may be sent. If data are owned by a third party, please indicate how others may request data access.

3. Please include captions for your Supporting Information files at the end of your manuscript, and update any in-text citations to match accordingly. Please see our Supporting Information guidelines for more information: http://journals.plos.org/plosone/s/supporting-information.

4. If the reviewer comments include a recommendation to cite specific previously published works, please review and evaluate these publications to determine whether they are relevant and should be cited. There is no requirement to cite these works unless the editor has indicated otherwise.

Additional Editor Comments (if provided):

Our reviewers have done a thorough review of the manuscript.

Authors have the privilege to submit their responses together with the necessary remedies or corrections as suggested/required. This will help the journal to make a final decision moving forward with this manuscript submission.

[Note: HTML markup is below. Please do not edit.]

Reviewers' comments:

Reviewer's Responses to Questions

**Comments to the Author**

1. Is the manuscript technically sound, and do the data support the conclusions?

Reviewer #1: Partly

Reviewer #2: Yes

Reviewer #3: Partly

**********

2. Has the statistical analysis been performed appropriately and rigorously?

Reviewer #1: Yes

Reviewer #2: Yes

Reviewer #3: N/A

**********

3. Have the authors made all data underlying the findings in their manuscript fully available?

The PLOS Data policy

Reviewer #1: Yes

Reviewer #2: Yes

Reviewer #3: No

**********

4. Is the manuscript presented in an intelligible fashion and written in standard English?

Reviewer #1: Yes

Reviewer #2: Yes

Reviewer #3: Yes

**********

Reviewer #1: 1. Is the manuscript technically sound, and do the data support the conclusions?

Partly. The study employs a recognized qualitative methodology (descriptive phenomenology with Braun & Clarke thematic analysis), and the seven themes are grounded in participant quotations. However, several technical concerns weaken the manuscript.

The authors claim a Husserlian transcendental phenomenological approach (line 162) but then apply Braun & Clarke's thematic analysis, which is epistemologically flexible and not inherently phenomenological. A true Husserlian analysis would typically use methods like Giorgi's or Colaizzi's procedures to arrive at essential structures of experience. The paper never produces phenomenological descriptions of essences; it produces a thematic codebook. The authors should either reframe the study as a generic qualitative descriptive study with thematic analysis or demonstrate how phenomenological reduction actually shaped the analytic steps beyond simply "bracketing."

The claim that "data saturation was reached" (line 187, 244) is asserted but never operationalized. The authors do not describe how saturation was assessed for instance, whether they tracked new codes per interview or used a stopping rule. Given the heterogeneity of participants (apprentice through master, self-employed through large-company, residential through industrial), 23 interviews may or may not have been sufficient, but the reader has no evidence either way.

The conclusions that a "self-reinforcing cycle" exists and that "joint effort" is needed are reasonable at a general level but are stated more strongly than the data warrant. The study documents co-occurrence of problems but does not establish causal or reinforcing relationships. The term "cycle" implies a dynamic model that the cross-sectional interview data cannot substantiate.

2. Has the statistical analysis been performed appropriately and rigorously?

Not applicable in the traditional sense, as this is a qualitative study. However, several quasi-quantitative claims need scrutiny. The authors report "68 reported incidents" and "approximately 2.956 incidents per person" (line 386), which implies precise counting from narrative data. No intercoder reliability metrics are provided for this classification exercise. The incident categories in Table 4 appear to overlap (e.g., "sewer-related staph infection" appears under physical injuries, while "walking pneumonia from exposure" is under occupational health risk), and the boundary rules are not defined. The percentages in Table 2 do not sum correctly within categories (e.g., Employment Type sums exceed 100% without explanation, which is likely because participants can be both "owner" and "self-employed," but this is never clarified). The decimal precision reported (e.g., 4.345%, 39.105%, 47.795%) is spurious given n=23 and should be rounded.

3. Is the manuscript presented in an intelligible fashion and written in standard English?

The manuscript is generally understandable but contains numerous grammatical, stylistic, and formatting issues that would need correction.

Specific examples include: sentence fragments and run-ons (e.g., line 67: "Despite these deaths or injuries, there are no studies that discuss the topics at hand", this claim is both syntactically awkward and overly broad); inconsistent table numbering (the interview guide is labeled "Table 1" in the text at line 199 but "Table 2" in its header; participant characteristics are labeled "Table 2" in the text at line 252 but appear to be the actual Table 1); verb tense shifts within paragraphs (e.g., lines 226–233 shift between past and present); missing articles and prepositions throughout; and a reference that includes a Chrome extension URL path (lines 617–619), which is not a valid citation format. The phrase "Participants comprised of" (line 174) should be "Participants comprised" or "Participants consisted of." Several participant accounts in the Results section read more like case summaries than analytic interpretations grounded in thematic structure.

The writing would benefit from professional copyediting before resubmission.

4. General Comments to the Authors

This study addresses an understudied population within the construction trades, and the authors' practical expertise in plumbing and OSHA training is a genuine asset. The topic is timely and relevant. However, several substantive issues should be addressed before the manuscript is suitable for publication.

First, the methodological framing needs clarification. The tension between claiming a Husserlian phenomenological approach and executing a standard thematic analysis undermines credibility. If the goal was to understand the "essence" of plumbers' safety experiences, the analysis should have produced phenomenological descriptions rather than a thematic codebook. Either re-align the methodology label with what was actually done (qualitative description with thematic analysis is perfectly respectable) or demonstrate how phenomenological reduction shaped each analytic phase.

Second, the dual role of the interviewer (30-year plumber, OSHA instructor) is acknowledged but insufficiently managed. The reflexive journal is mentioned twice but never excerpted or discussed in the results. How did the interviewer's expertise shape follow-up probing? Were there moments where bracketing failed? Transparency here would strengthen, not weaken, the paper.

Third, the results section would benefit from more analytic depth and less descriptive cataloguing. Currently, each theme is presented as a series of participant-by-participant incident summaries. Stronger qualitative work would identify patterns across participants, explore contradictions, and build interpretive arguments. For example, the "get-it-done culture" is a rich finding but is discussed almost entirely in the Discussion rather than being developed as a cross-cutting analytic thread in the Results.

Fourth, the theoretical framing is thin. The abstract mentions "structural, cultural, and behavioral lenses," and several theories are referenced in passing (Bandura's social cognitive theory, Rogers' diffusion of innovations, Rosenstock's health belief model, Scott's institutional theory all in the reference list), but none are systematically applied in the analysis or discussion. Selecting one or two frameworks and using them to organize the interpretation would add considerable depth.

Fifth, several specific claims need supporting evidence or qualification. The statement that "there are no studies that discuss the topics at hand" (line 68) is too sweeping, the authors themselves cite several relevant studies. The claim about "3 fatalities" in Table 4 needs clarification: were these experienced directly by participants, witnessed, or reported secondhand? This distinction matters for data quality. The contributing factors table (Table 6) lists factors without frequencies, despite the header promising them.

Sixth, the interview guide (Table 1/2) shows different question sets for employees and owners, but the analysis does not systematically compare these two groups. Given that 6 participants were owners and 17 were employees, this contrast could yield important insights about the "perception gap" the authors discuss.

Seventh, member checking or respondent validation is not mentioned. While not mandatory, some form of credibility check beyond the research team (e.g., returning summaries to participants, consulting an external auditor) would strengthen trustworthiness.

Finally, minor but notable: the paper lists "Osburn, Woo Lee, and Gambatese, 2022" in line 69 but this reference does not appear in the reference list. Several other in-text citations (e.g., Avetta 2025, NIOSH 2018) need verification against the reference list for consistency.

In summary, the manuscript tackles an important gap and offers valuable practitioner-informed data, but it needs methodological tightening, deeper analysis, better alignment between claims and evidence, and language editing before it meets the standard for publication.

Reviewer #2: Abstract

The abstract addresses a worthwhile problem, but it would benefit from stronger precision and clearer internal structure. The background raises an important question. However, the specific research gap is not clearly articulated before the study objective is introduced. In addition, the methods, results, and conclusions are compressed into long sentences, and the abstract lists the seven themes without identifying which systemic issues were most recurrent or most analytically important. The final claim is also broader than the evidence presented in a sample of 23 plumbers can fully support (see p. 7, lines 35–58). To resolve these issues, the author or authors should consider adding one or two sentences to:

a. state the specific research gap that gave rise to the study before presenting the aim and objectives. The author (s) can state that limited qualitative research has examined why existing safety measures continue to fail plumbers, especially in trade-specific U.S. contexts such as Texas (see p. 7, lines 35–45). This is only a suggestion. The author (s) can revise this aspect according to their needs;

b. separate the objective, methods, and results more clearly and identify the most recurrent systemic barriers, rather than only listing all seven themes in one sequence (see p. 7, lines 41–55); and

c. temper the concluding claim so that it remains proportionate to a qualitative sample of 23 participants and does not imply wider generalisability than the design allows (see p. 7, lines 56–58).

Introduction Section

The introduction establishes the occupational relevance of plumbing, but the problem framing still requires sharpening. In particular, the statement that no studies address the topic is overly absolute, and the section moves from a general hazard description to the study aim without sufficiently distinguishing what is already known from what remains underexplored. The Texas justification is useful, but the novelty of the study as trade-specific, qualitative, and system-oriented should be stated more directly and earlier (see p. 8, lines 64–80; pp. 9–11, lines 81–138). To resolve these issues, the author or authors should consider adding one or two sentences to:

a. replace absolute statements such as the claim that no studies exist with a more accurate formulation showing that plumber-specific qualitative research remains limited compared with broader construction safety studies (see p. 8, lines 67–69);

b. differentiate more clearly between general construction hazard literature and the specific knowledge gap concerning plumbers, trade-specific exposures, and organisational safety failures (see pp. 8–10, lines 64–125); and

c. state the study’s significance and novelty more directly by explaining that it brings worker-centred qualitative evidence to a field often dominated by administrative statistics or broader construction analyses (see p. 11, lines 126–138).

The author or authors should consider reading and citing this peer-reviewed article: “Assessing plumbing standards compliance, challenges and training effectiveness in the Ghanaian construction industry: an institutional and compliance theory perspective”. International Journal of Building Pathology and Adaptation, 1–21. https://doi.org/10.1108/IJBPA-05-2025-0103

Methods and research design

The methodology section contains useful details. However, it still needs stronger internal consistency and clearer justification. Most importantly, the consent procedure is reported inconsistently: the submission metadata states electronic written consent, whereas the methods section states verbal consent. The section also describes the study as descriptive phenomenology but analyses the data using Braun and Clarke’s thematic analysis, a combination that is not fully justified. In addition, the recruitment, saturation, and trustworthiness procedures, as well as the role of interviewer positionality, need fuller explanation (see pp. 11–16, especially p. 11, lines 141–148; pp. 13–16, lines 173–245; p. 14, lines 190–200). To resolve these issues, the author or authors should consider adding one or two sentences to:

a. reconcile the form of consent reported across the manuscript and submission materials, and state consistently whether consent was electronic written consent, verbal consent, or both (see p. 6, lines 23–26; p. 11, lines 145–148);

b. justify more clearly how descriptive phenomenology aligns with the use of Braun and Clarke’s thematic analysis, or revise the methodological positioning if the study is more accurately qualitative descriptive or reflexive thematic in practice (see p. 12, lines 161–170; p. 15, lines 224–245); and

c. explain more explicitly how recruitment occurred, why 23 participants were sufficient, how saturation was operationalised, and what steps were taken to enhance trustworthiness, such as peer review of coding, audit trails, or member checking if used (see pp. 13–16, lines 173–245).

Results

The results section contains rich qualitative material. However, its presentation needs clearer analytical control. First, the participant-characteristics table is mislabelled, and the table numbering becomes confusing. Second, the section mixes personal injuries, witnessed coworker incidents, near misses, and occupational health risks in ways that are not always analytically distinguished. Third, some descriptive counts and recommendations are presented without a clear explanation of how they were derived from the thematic analysis or how they support the main qualitative argument. This reduces transparency and may blur the boundary between theme-based interpretation and incident enumeration (see pp. 17–27, especially p. 17, lines 249–253; pp. 24–27, lines 367–413). To resolve these issues, the author or authors should consider adding one or two sentences to:

a. correct the table numbering and captions so that the participant table and interview-guide table are accurately labelled and consistently referenced (see p. 14, lines 190–200; p. 17, lines 249–253);

b. distinguish more clearly between self-experienced injuries, witnessed coworker incidents, near misses, and occupational health risks so the reader can see exactly what the evidence base represents (see pp. 24–25, lines 367–389); and

c. explain more explicitly how the frequency counts, contributing factors, and participant recommendations were generated from the qualitative dataset and how they complement, rather than replace, the thematic analysis (see pp. 26–27, lines 391–413).

Discussion Section

The discussion is relevant and practically oriented. However, parts of it remain too confirmatory and occasionally move beyond the immediate evidence. In several places, the section relies heavily on external literature to explain the findings, while the distinct contribution of the present interview data is not always sufficiently foregrounded. The discussion of a “perception gap” between plumbers and employers is potentially important, but it should be tied more explicitly to the owner-versus-employee evidence generated in this study. Some recommendations are also broader than the data directly support (see pp. 28–30, lines 415–479). To resolve these issues, the author or authors should consider adding one or two sentences to:

a. distinguish more carefully between what is directly supported by participants’ accounts and what is being inferred from the wider literature on construction and high-risk occupations (see pp. 28–29, lines 415–467);

b. compare the findings more critically with plumber-specific and trade-specific safety literature, rather than relying mainly on broader construction safety studies for confirmation (see pp. 28–30, lines 424–475); and

c. show more explicitly how the reported owner and employee perspectives support the argument about perceptual or organisational gaps, so that this interpretation remains clearly grounded in the study data (see pp. 17–18, participant characteristics; pp. 28–30, lines 460–467).

The author or authors should consider reading and citing this peer-reviewed article: “Assessing plumbing standards compliance, challenges and training effectiveness in the Ghanaian construction industry: an institutional and compliance theory perspective”. International Journal of Building Pathology and Adaptation, 1–21. https://doi.org/10.1108/IJBPA-05-2025-0103

Reviewer #3: 1. In the abstract, the initial part of the Objectives is methodology and not objectives.

2. On line 179, the table number stated is not correct.

3. The authors did not explain how the participants of the study were selected.

4. The authors did not explain how they obtained the participants of the study’s information/contact which was used for reaching out to them for the interview.

5. The interview guide provided (table 1), did not contain anonymity and confidentiality statement.

6. The interview guide provided (table 1), contained just few questions. I am not sure if those questions could generate all the responses presented in the Results.

7. How was information in Table 2 obtained? As the interview guide did not elicit that information.

8. Table 7 caption is not complete. Factors of what?

9. Recommendations of the study must be provided.

**********

what does this mean?). If published, this will include your full peer review and any attached files.

If you choose “no”, your identity will remain anonymous but your review may still be made public.

**Do you want your identity to be public for this peer review?**  For information about this choice, including consent withdrawal, please see our Privacy Policy

Reviewer #1: **Yes:**  Henry Kofi Dansu

Reviewer #2: **Yes:**  Frederick Owusu Danso

Reviewer #3: No

**********

[NOTE: If reviewer comments were submitted as an attachment file, they will be attached to this email and accessible via the submission site. Please log into your account, locate the manuscript record, and check for the action link "View Attachments". If this link does not appear, there are no attachment files.]

To ensure your figures meet our technical requirements, please review our figure guidelines: https://journals.plos.org/plosone/s/figures

You may also use PLOS’s free figure tool, NAAS, to help you prepare publication quality figures: https://journals.plos.org/plosone/s/figures#loc-tools-for-figure-preparation.

NAAS will assess whether your figures meet our technical requirements by comparing each figure against our figure specifications.

# Author comment: Author response to Decision Letter 1

**DOI:** 10.1371/journal.pone.0356512.r002

9 Jul 2026

RE: Response to Reviewers: “Occupational Safety and Health Risks Among Plumbers: A Qualitative Study of Workplace Hazards, Safety Practices, and System-Level Gaps in Texas”

Dear Editor and Reviewers,

We thank the Academic Editor and all three reviewers for their careful and constructive engagement with our manuscript. We are grateful for such a thorough reading of our manuscript. Their comments prompted a substantial revision: we reframed our methodological approach, added cross-participant analytic synthesis throughout the Results, grounded our discussion of the owner/employee perception gap in the study's own sample, and corrected a number of reporting and citation errors. We address each point below, with section numbers referring to the revised submission.

Reviewer #1

Husserlian phenomenology vs. Braun & Clarke thematic analysis. The reviewer is correct that the original framing did not match the analysis performed. The study is now described throughout as a qualitative descriptive design using Braun and Clarke's (2006, 2019) reflexive thematic analysis; the Husserlian/transcendental phenomenology language has been removed from §2.3, along with the now-uncited Husserl (1970) reference. The researcher's reflexive bracketing practice was retained, as it fits comfortably within reflexive thematic analysis.

Data saturation not operationalized. Agreed. §2.5 now states the stopping rule that was actually used: new codes were tracked per interview, and data collection continued until several consecutive transcripts introduced nothing new.

"Self-reinforcing cycle" and "joint effort" overstate what a cross-sectional design can show. We have softened this language in the Abstract, Discussion, and Limitations, describing the problems documented as co-occurring rather than causally reinforcing.

Precision of "68 incidents" and "2.956 incidents per person"; missing intercoder reliability. The per-person figure is rounded to approximately 3.0. We have also added a direct disclosure that coding was conducted by a single researcher, with the research team's Zoom discussions functioning as peer debriefing rather than independent double-coding, and that no formal reliability statistic was calculated (§2.7 and §6).

Table 4 category overlap (e.g., staph infection under physical injuries vs. walking pneumonia under occupational health risk). This was a fair point. We applied a consistent classification rule going forward: incidents originating from a discrete mechanical or traumatic event are physical injuries; illnesses or infections arising primarily from exposure are occupational health risks. The sewer-related staph infection and testicular infection have been moved into Occupational Health Risk under this rule, with counts recalculated (Physical injuries n=41; Occupational Health Risk n=17; total unchanged at 68).

Percentages that don't sum correctly; spurious decimal precision. Table 2 percentages are now rounded to one decimal. We also split the former "Employment Type" variable into two independent groupings, Business Structure (self-employed/small company/large company) and Role (owner/employee), with a footnote noting the two can overlap for a given participant.

Grammar, table mislabeling, citation formatting. "Participants comprised of" has been corrected to "Participants comprised"; the interview guide and participant-characteristics tables are now numbered and cross-referenced consistently as Table 1 and Table 2; and the broken chrome-extension:// URL in the Yi (2023) reference now points to a standard URL.

Interviewer's dual role and an unexcerpted reflexive journal. We appreciate the push for transparency here. §2.6 now includes three direct excerpts from the researcher's reflexive journal: two showing moments where OSHA/plumbing expertise prompted an urge to probe further that the researcher consciously held back, and a third showing restraint driven by sensitivity to a participant's injury rather than professional judgment.

Results read as participant-by-participant cataloging rather than analytic argument. We restructured §3.2 to add cross-participant synthesis to all seven themes — for instance, noting that PPE noncompliance spans experience levels rather than concentrating among newer workers, distinguishing behavioral from maintenance-driven tool-use incidents, separating acute from cumulative health-exposure profiles, and showing that policy-enforcement gaps appear at both ends of company size.

Thin theoretical framing. We have taken up Bandura's (1986) social cognitive theory as an organizing lens for the cultural and behavioral themes in the Discussion, applying it directly to the "get-it-done" culture findings (observational learning, reciprocal determinism) rather than leaving it as an uncontextualized citation.

"There are no studies that discuss the topics at hand" is too sweeping. Revised to state that plumber-specific qualitative research remains limited relative to the broader construction safety literature. We also identified that Osburn, Lee, and Gambatese (2022) was cited in-text but missing from the reference list; it has been added.

Table 4's three fatalities — witnessed, experienced, or secondhand? Clarified: two are labeled witnessed directly, and the trench cave-in is labeled reported secondhand, from a different company than the participant's own.

Owner/employee split (6 owners, 17 employees) not systematically compared. §3.2 now includes a comparative analysis across all six owner participants, examining how ownership itself removes a layer of oversight and accountability (no supervisor to report to, competitive cost pressure, the capacity limits of a lean operation), even as several owners describe enforcing stricter policies than they experienced as employees.

No mention of member checking. Correct — none was conducted. §6 now states this plainly, noting that peer debriefing during theme refinement served as the primary credibility check.

Table 6 promises frequencies it doesn't provide. The lead-in sentence has been corrected to remove that claim.

Avetta (2025) and NIOSH (2018) citation consistency. Avetta (2025) is now cited in-text alongside Neubauer et al. (2019) in the perception-gap discussion. NIOSH (2018) was already cited correctly and matches the reference list.

Reviewer #2

Abstract lacks a stated gap, blends objective/methods/results, and overstates its conclusion. The Abstract now states the research gap before the aim, separates Objectives/Methods/Results/Conclusion more cleanly, names the most analytically central themes rather than listing all seven with equal weight, and tempers the concluding claim to the scope a 23-participant qualitative sample can support.

Introduction's absolute claim and thin differentiation from general construction literature. The overbroad "no studies" claim has been replaced with a more precise statement of the gap; we have also strengthened our use of plumbing-specific comparators in the Discussion (see next point).

Suggested Ghana IJBPA article (Danso et al., 2025). Added to the reference list and cited in-text in the Discussion, where its institutional and compliance theory findings help explain why weak enforcement, rather than the absence of standards, appears to drive plumbing-safety noncompliance.

Inconsistent consent reporting (electronic written vs. verbal). Reconciled — the manuscript now describes verbal consent consistently throughout.

Descriptive phenomenology paired with Braun & Clarke thematic analysis, unjustified. Addressed as described under Reviewer #1, point 1: the methodological label now matches the analysis performed.

Recruitment, saturation, and trustworthiness underexplained. §2.4–2.5 now describe recruitment as drawing on a dataset of licensed Texas plumbers who had completed continuing education courses, contacted via a distributed recruitment statement. Saturation tracking is operationalized as described under Reviewer #1, point 2, and the absence of member checking is now stated directly rather than left silent.

Participant table mislabeled; injury types not analytically distinguished; frequencies unexplained. Table numbering is corrected throughout; the physical-injury/occupational-health-risk boundary is clarified and reapplied as described under Reviewer #1, point 5; and the Table 6 frequency claim has been corrected.

Discussion leans on general literature over the study's own data; perception gap not tied to owner/employee data. We now distinguish more clearly between claims grounded in participant accounts and claims drawn from the wider literature, bring in Danso et al. (2025) and Mbada et al. (2022) as plumbing-specific comparators, and ground the perception-gap discussion directly in the owner-versus-employee comparison described above.

Reviewer #3

Abstract's Objectives section opens with methodology, not the aim. Corrected — the Objectives sentence now states the research aim and gap without embedding method-level detail.

Table numbering error (line 179). Corrected; Table 1 (Interview Guide) and Table 2 (Participant Characteristics) are now numbered and referenced consistently.

Comments 3–4: How participants were selected and how contact information was obtained. §2.4 now states that recruitment drew on a dataset of licensed Texas plumbers who had completed continuing education courses, with a recruitment statement distributed to that group.

Comment 5: Interview guide lacks an anonymity/confidentiality statement. Added as the opening item of the interview guide (Table 1).

Comment 6: Interview guide seems too brief to generate the range of Results reported. The employee and owner question sets differ in depth by design — the employee protocol was built for detailed incident-level narrative, since employees perform the frontline tasks under study, while the owner protocol focused on management-level oversight. The semi-structured format allowed organic follow-up beyond the printed items, which accounts for the richer detail reported in Results.

Comment 7: How was Table 2's information obtained, given the guide doesn't ask for it directly? §3.1 now states that years of experience and employment classification were elicited through organic follow-up during the interview rather than the fixed guide questions.

Comment 8: Table 7 caption incomplete ("Factors" of what?). Corrected to "Table 7: Participant Recommendations for Improving Safety," matching §3.6.

Comment 9: Study recommendations must be provided. A new researcher-authored Recommendations section (§5) has been added, distinct from the participant-reported recommendations in Table 7, addressing training access, equipment-maintenance protocols, consistent policy enforcement, joint owner-employee safety planning, and insurance or bidding incentives for documented safety investment.

We thank the reviewers again for pushing us toward a more rigorous and transparent manuscript.

Grateful,

Mary Conger, MS, and Avishek Choudhury, PhD

Attachment Submitted filename: Response to Reviewers.docx

# Peer review: Decision Letter 1

**DOI:** 10.1371/journal.pone.0356512.r003

**Authors:** Putri Anis Syahira Mohamad Jamil

4 Aug 2026

Occupational Safety and Health Risks Among Plumbers: A Qualitative Study of Workplace Hazards, Safety Practices, and System-Level Gaps in Texas

PONE-D-26-20219R1

Dear Dr. Choudhury,

We’re pleased to inform you that your manuscript has been judged scientifically suitable for publication and will be formally accepted for publication once it meets all outstanding technical requirements.

Within one week, you’ll receive an e-mail detailing the required amendments. When these have been addressed, you’ll receive a formal acceptance letter and your manuscript will be scheduled for publication.

An invoice will be generated when your article is formally accepted. Please note, if your institution has a publishing partnership with PLOS and your article meets the relevant criteria, all or part of your publication costs will be covered. Please make sure your user information is up-to-date by logging into Editorial Manager at Editorial Manager® and clicking the ‘Update My Information' link at the top of the page. For questions related to billing, please contact billing support.

If your institution or institutions have a press office, please notify them about your upcoming paper to help maximize its impact. If they’ll be preparing press materials, please inform our press team as soon as possible -- no later than 48 hours after receiving the formal acceptance. Your manuscript will remain under strict press embargo until 2 pm Eastern Time on the date of publication. For more information, please contact onepress@plos.org.

Kind regards,

Putri Anis Syahira Mohamad Jamil

Academic Editor

PLOS One

Additional Editor Comments (optional):

Reviewers' comments:

Reviewer's Responses to Questions

**Comments to the Author**

Reviewer #1: All comments have been addressed

Reviewer #2: All comments have been addressed

**********

2. Is the manuscript technically sound, and do the data support the conclusions?

Reviewer #1: Yes

Reviewer #2: Yes

**********

3. Has the statistical analysis been performed appropriately and rigorously?

Reviewer #1: Yes

Reviewer #2: Yes

**********

4. Have the authors made all data underlying the findings in their manuscript fully available?

The PLOS Data policy

Reviewer #1: Yes

Reviewer #2: Yes

**********

5. Is the manuscript presented in an intelligible fashion and written in standard English?

Reviewer #1: Yes

Reviewer #2: Yes

**********

Reviewer #1: (No Response)

Reviewer #2: Please, the author or authors have addressed all the issues raised and I have recommended acceptance. Congratulations to the author or authors

**********

what does this mean?). If published, this will include your full peer review and any attached files.

If you choose “no”, your identity will remain anonymous but your review may still be made public.

**Do you want your identity to be public for this peer review?**  For information about this choice, including consent withdrawal, please see our Privacy Policy

Reviewer #1: **Yes:**  Henry Kofi Dansu

Reviewer #2: No

**********

# Editor report: Acceptance letter

**DOI:** 10.1371/journal.pone.0356512.r004

**Authors:** Putri Anis Syahira Mohamad Jamil

PONE-D-26-20219R1

PLOS One

Dear Dr. Choudhury,

I'm pleased to inform you that your manuscript has been deemed suitable for publication in PLOS One. Congratulations! Your manuscript is now being handed over to our production team.

At this stage, our production department will prepare your paper for publication. This includes ensuring the following:

* All references, tables, and figures are properly cited

* All relevant supporting information is included in the manuscript submission,

* There are no issues that prevent the paper from being properly typeset

You will receive further instructions from the production team, including instructions on how to review your proof when it is ready. Please keep in mind that we are working through a large volume of accepted articles, so please give us a few days to review your paper and let you know the next and final steps.

Lastly, if your institution or institutions have a press office, please let them know about your upcoming paper now to help maximize its impact. If they'll be preparing press materials, please inform our press team within the next 48 hours. Your manuscript will remain under strict press embargo until 2 pm Eastern Time on the date of publication. For more information, please contact onepress@plos.org.

You will receive an invoice from PLOS for your publication fee after your manuscript has reached the completed accept phase. If you receive an email requesting payment before acceptance or for any other service, this may be a phishing scheme. Learn how to identify phishing emails and protect your accounts at https://explore.plos.org/phishing.

If we can help with anything else, please email us at customercare@plos.org.

Thank you for submitting your work to PLOS ONE and supporting open access.

Kind regards,

PLOS ONE Editorial Office Staff

on behalf of

Dr. Putri Anis Syahira Mohamad Jamil

Academic Editor

PLOS One
